# Targeting HECTD3-IKKα axis inhibits inflammation-related metastasis

**DOI:** 10.1038/s41392-022-01057-0

**Published:** 2022-08-03

**Authors:** Fubing Li, Huichun Liang, Hua You, Ji Xiao, Houjun Xia, Xi Chen, Maobo Huang, Zhuo Cheng, Chuanyu Yang, Wenjing Liu, Hailin Zhang, Li Zeng, Yingying Wu, Fei Ge, Zhen Li, Wenhui Zhou, Yi Wen, Zhongmei Zhou, Rong Liu, Dewei Jiang, Ni Xie, Bin Liang, Zhenzhen Liu, Yanjie Kong, Ceshi Chen

**Affiliations:** 1 Key Laboratory of Animal Models and Human Disease Mechanisms of the Chinese Academy of Sciences and Yunnan Province, KIZ-CUHK Joint Laboratory of Bioresources and Molecular Research in Common Diseases, Kunming Institute of Zoology, Chinese Academy of Sciences, Kunming, 650223 China; 2 Affiliated Cancer Hospital & Institute of Guangzhou Medical University, Guangzhou, 510095 China; 3 Department of Pathology, School of Basic Medicine, Yunnan University of Chinese Medicine, Kunming, 650500 China; 4 College of Life Science and Technology, Guangzhou Jinan Biomedicine Research and Development Center, Jinan University, Guangzhou, 510632 China; 5 Center for Cancer Immunology, Institute of Biomedicine and Biotechnology, Shenzhen Institute of Advanced Technology, Chinese Academy of Sciences, Shenzhen, 518055 China; 6 First Affiliated Hospital of Kunming Medical University, Kunming, Yunnan 650032 China; 7 Department of the Third Breast Surgery, the Third Affiliated Hospital of Kunming Medical University, Kunming, Yunnan 650118 China; 8 Hubei Key Laboratory of Embryonic Stem Cell Research, Hubei University of Medicine, Shiyan, 442000 China; 9 Biobank, Shenzhen Second People’s Hospital, the First Affiliated Hospital of Shenzhen University, Health Science Center, Shenzhen, 518035 China; 10 Center for Life Sciences, School of Life Sciences, Yunnan University, Kunming, Yunnan 650091 China; 11 Department of Breast disease, Henan Breast Cancer Center, Affiliated Cancer Hospital of Zhengzhou University & Henan Cancer Hospital, Zhengzhou, 450008 China

**Keywords:** Metastasis, Breast cancer

## Abstract

Metastasis is the leading cause of cancer-related death. The interactions between circulating tumor cells and endothelial adhesion molecules in distant organs is a key step during extravasation in hematogenous metastasis. Surgery is a common intervention for most primary solid tumors. However, surgical trauma-related systemic inflammation facilitates distant tumor metastasis by increasing the spread and adhesion of tumor cells to vascular endothelial cells (ECs). Currently, there are no effective interventions to prevent distant metastasis. Here, we show that HECTD3 deficiency in ECs significantly reduces tumor metastasis in multiple mouse models. HECTD3 depletion downregulates expression of adhesion molecules, such as VCAM-1, ICAM-1 and E-selectin, in mouse primary ECs and HUVECs stimulated by inflammatory factors and inhibits adhesion of tumor cells to ECs both in vitro and in vivo. We demonstrate that HECTD3 promotes stabilization, nuclear localization and kinase activity of IKKα by ubiquitinating IKKα with K27- and K63-linked polyubiquitin chains at K296, increasing phosphorylation of histone H3 to promote NF-κB target gene transcription. Knockout of HECTD3 in endothelium significantly inhibits tumor cells lung colonization, while conditional knockin promotes that. IKKα kinase inhibitors prevented LPS-induced pulmonary metastasis. These findings reveal the promotional role of the HECTD3-IKKα axis in tumor hematogenous metastasis and provide a potential strategy for tumor metastasis prevention.

## Introduction

Metastasis accounts for 90% of deaths in cancer patients.^1^ Cancer patients without clinical symptoms after initial treatment frequently develop distant metastasis years later.^2^ Surgery is a common early intervention for most solid tumors. However, mechanical trauma and the subsequent wound healing process constitute favorable factors for metastasis through several mechanisms, including release of circulating tumor cells (CTCs)^3,4^ and triggering systemic inflammation.^5^ To avoid anoikis during metastasis, CTCs must attach to the vasculature of distant organs and extravasate into the perivascular tissue.

Accumulating data indicate that systemic inflammation potentiates the adhesion of CTCs to vascular endothelial cells (ECs) of distant organs. This is a key step of extravasation in hematogenous metastasis.^5^ CTC extravasation typically occurs in small capillaries, where cancer cells are arrested by the endothelium via interaction with a wide range of adhesion molecules of ECs, including E-selectin, ICAM-1 (intercellular-adhesion molecule-1) and VCAM-1 (vascular cell adhesion molecule-1), through their cognate ligands.^6,7^ E-selectin is expressed exclusively by ECs in rapid response to inflammatory stimuli (e.g., TNF-α and IL-1β). E-selectin recognizes various glycoprotein ligands expressed on cancer cells, including a specific sialofucosylated glycoform of CD44, PSGL1, CD24, MUC1 and LGALS3BP. Cancer cell interaction with E-selectin seems to be the initial step for CTC extravasation and is essential for metastasis.^8^ It has been reported that bone vascular E-selectin directly captures breast cancer cells to promote bone metastasis.^9^ Consistently, atrial natriuretic peptide (ANP) prevents cancer metastasis by suppressing E-selectin expression by ECs.^10^ Subsequently, ICAM-1 and VCAM-1 on ECs allow adhesion of cancer cells to ECs. ICAM-1 forms Y-shaped covalent homodimers at the cell surface, which forcefully bind to the abnormal glycoform of MUC1 associated with cancer cells.^11^ VCAM-1 is expressed on the luminal and lateral side of ECs in response to inflammatory factors. VCAM-1 also increases the adhesion of various subsets of leukocytes^12^ and tumor cells^13^ via recognition of integrins, such as VLA-4 (late activation antigen-4) or integrin α4. Pretreatment of inflammatory factors, such as TNFα, IL-1β and SDF-1, or exposure to surgical stress or sepsis^14^ increased VCAM-1 expression on pulmonary ECs of mice, leading to increased numbers of lung metastatic nodules after intravenous injection of tumor cells.^15^

E-selectin,^16^ ICAM-1^17^ and VCAM-1^18^ are NF-κB target genes in ECs. When endothelial cells receive inflammatory stimuli, such as TNFα or lipopolysaccharide (LPS), the IKK kinase complex, containing IKKα, IKKβ and NEMO (IKKγ), phosphorylates IκBα and targets IκBα for proteasomal degradation. Free from IκBα binding, the NF-κB dimer p50/65 accumulates in the nucleus and binds to specific promoters to activate transcription of downstream target genes. Although IKKα and IKKβ have similar structures, they exhibit differential regulatory patterns.^19,20^ IKKα is dispensable for IκBα degradation,^21^ but IKKα promotes processing of the p100 precursor into p52 in the noncanonical NF-κB pathway.^22^ Additionally, IKKα harbors a specific nuclear localization signaling and can directly regulate NF-κB-dependent gene transcription in the nucleus. Nuclear IKKα is recruited to NF-κB binding chromatin and phosphorylates histone H3 at Ser10 to activate NF-κB target gene transcription.^23,24^

HECTD3 is a HECT-type E3 ubiquitin ligase with multiple substrates and functions.^25^ HECTD3 confers chemotherapy drug resistance by ubiquitinating MALT1,^26^ Caspase-8,^27^ and Caspase-9.^28^ Hectd3 promoted pathogenic Th17 cell generation by ubiquitinating MALT1 and STAT3 in an experimental autoimmune encephalomyelitis (EAE) mouse model.^29^ Our recent study suggested that Hectd3 promotes type I interferon production and intracellular bacterial infection by increasing K63-linked polyubiquitination of TRAF3.^30^ In most situations, HECTD3 does not target its substrates for degradation because the ubiquitination chains mediated by HECTD3 are not linked through K48 or K11. Instead, HECTD3 protects MALT1 from degradation in response to chemotherapy.^26^ However, the role of HECTD3 in cancer metastasis has not been previously reported.

In this study, we utilized multiple mouse models to examine the function of HECTD3 in tumor metastasis and observed that HECTD3 promotes adhesion of tumor cells to the vascular endothelium by upregulating expression of adhesion molecules on ECs in response to inflammatory conditions, which promotes tumor hematogenous metastasis. The mechanism involves a process that HECTD3 ubiquitinates IKKα to promote its stability and nuclear kinase activity toward histone H3, eventually potentiating NF-κB-mediated gene transcription. We demonstrated that inhibition of the HECTD3-IKKα axis effectively inhibited tumor metastasis induced by systemic inflammation.

## Results

### Hectd3 deficiency inhibits inflammation-induced tumor metastasis in mice

**Fig. 1:**
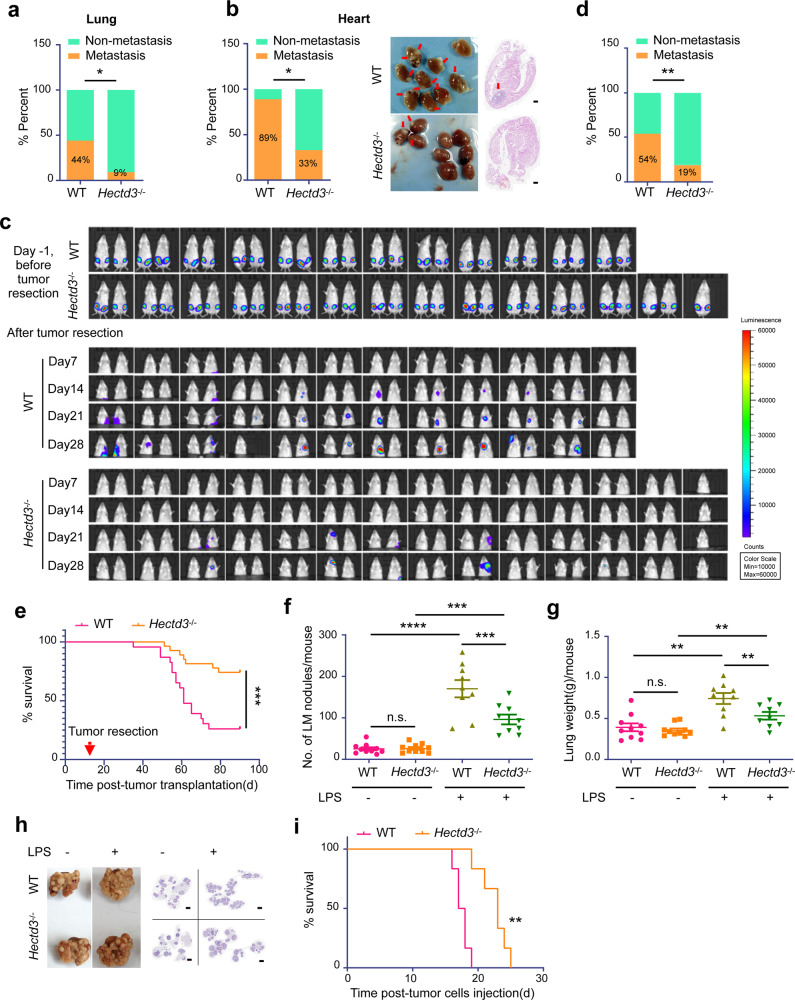
*Hectd3* knockout inhibits inflammation-induced tumor metastasis in mice.**a** A comparison of the incidence of lung metastases in WT (*n* = 9) versus *Hectd3*^−/−^ (*n* = 11) mice with an FVB genetic background. PyMT-induced tumor cells were orthotopically injected into the fat pad of both groups of mice (5 × 10^5^ cells per mouse). Primary tumors were removed 20 days later. Mice were sacrificed after 2 months, and the incidence of lung metastasis was recorded. **b** PyMT-induced breast tumor cells were inoculated as described above. The incidence of heart metastasis (left), representative heart metastasis nodule images and H&E staining (right) are shown. **c** 4T1-Luc2 cells were injected orthotopically into the fourth pair of fat pads of WT (*n* = 24) and *Hectd3*^−/−^ (*n* = 27) BALB/c mice (bilateral, 1 × 10^5^ cells per point). Eleven days after transplantation (Day -1), primary tumors were imaged using a bioluminescent IVIS system (upper). Twelve days after transplantation (Day 0), primary tumors were removed, then tumor metastasis was monitored weekly by imaging and representative bioluminescence images are shown (lower). **d** A comparison of the incidence of metastases in WT versus *Hectd3*^−/−^ mice from panel **c**. **e** Kaplan–Meier survival curves of WT (*n* = 24) and *Hectd3*^−/−^ (*n* = 27) mice which 4T1-Luc2 primary tumors were removed 12 days after transplantation. **f** WT and *Hectd3*^−/−^ FVB mice were intravenously injected with or without LPS (1 mg/kg). 5 h later, PyMT-induced breast tumor cells were injected through the tail vein (2 × 10^5^ cells per mouse). Each group contained 9–10 mice, and mice were sacrificed 20 days after injection of tumor cells. The graph shows the number of pulmonary metastasis nodules in each group of mice. **g** The weight of the whole lung with metastatic nodules in each group of mice from panel **f**. **h** Representative lung metastasis nodule images and corresponding H&E staining of the lungs in different groups of mice from panel **f**. **i** Kaplan–Meier survival curves of WT (*n* = 6) mice and *Hectd3*^−/−^ (*n* = 6) mice pretreated with LPS and transplanted with PyMT-induced breast tumor cells through tail vein. Data represent three independent experiments for all of the above experiSments. Data are presented as the mean ± SEM, and statistics were calculated using the Chi-square test for **a**, **b** and **d**, two-way ANOVA for **f**, **g**, and log-rank test for **e** and **i**. *P < 0.05; **P < 0.01; ***P < 0.001; n.s., not significant. Scale bars are 500 μm for **b** and 2 mm for **h**

To determine the role of Hectd3 in tumor metastasis, we analyzed metastasis susceptibility in Hectd3-deficient mice utilizing two malignant mouse breast cancer metastasis models after surgery. We generated PyMT-induced mouse breast tumor cells by intraductal injection of lentivirus overexpressing PyMT into the mammary duct of wild type (WT) female FVB mice.^31^ Then, we transplanted PyMT-induced mouse breast tumor cells into WT and *Hectd3*^−/−^ mice and examined the lungs 2 months after resection of orthotopic tumors. Compared to WT mice, Hectd3 deficiency significantly inhibited lung metastasis (Fig. 1a) and heart metastasis (Fig. 1b). 4T1-Luc2 can spontaneously metastasize to multiple organs in BALB/c mice from the breast.^32,33^ We orthotopically inoculated 4T1-Luc2 cells into the fourth mammary fat pad of WT and *Hectd3*^−/−^ female BALB/c mice. Eleven days later, mice were imaged using a bioluminescent IVIS system, confirming that tumor size was consistent across animals between these two groups (Fig. 1c). On day 12, tumors were surgically removed. Actually, the volume of 4T1-Luc2 primary tumor was larger in *Hectd3*^−/−^ mice than in WT mice (Data not shown). Tumor metastasis was monitored weekly by imaging. We found that 4T1-Luc2 tumor metastasis was markedly suppressed in *Hectd3*^−/−^ mice (19%, 5/27), compared with that in WT mice (54%, 13/24) (Fig. 1c, d). In addition, Hectd3 deficiency significantly prolonged mouse survival (Fig. 1e), demonstrating that Hectd3 deficiency in the tumor microenvironment inhibits surgery-associated tumor metastasis.

As mentioned earlier, surgery may promote metastasis by increasing tumor cell dissemination and inducing systemic inflammation. In some tumor surgeries, >40% of patients develop peritonitis, pneumonia, sepsis, or severe postoperative infection, all of which can lead to recurrence and metastasis. Firstly, we detected several inflammation factors, such as LPS, TNFα, IL-1β and IL-6, in the sera of WT and *Hectd3*^−/−^ mice burdening primary tumor (0 h) or surgically removed primary tumor 6 h or 12 h later. Limulus amebocyte lysate assay and ELISA results showed that the serum LPS activity/level increased significantly in both WT and *Hectd3*^−/−^ mice 12 h after surgery, but there was no difference between WT and Hectd3 *Hectd3*^−/−^ mice (Supplementary Fig. 1a, b). ELISA results showed that the serum TNFα level increased 6 h and 12 h after surgery (Supplementary Fig. 1c), but the serum IL-1β and IL-6 levels had no significant change (Supplementary Fig. 1d, e). These results indicate that surgery induces inflammation by increasing LPS and TNFα levels in both WT and Hectd3 *Hectd3*^−/−^ mice.

To further investigate whether Hectd3 deficiency inhibits the metastasis of cancer cells to inflamed organs, we intravenously injected WT and *Hectd3*^−/−^ female FVB mice with LPS, which mimics systemic inflammation in response to surgical stress,^5,34^ followed by tail-vein injection of PyMT-induced mouse breast tumor cells. *Hectd3* knockout (KO) had no effect on the lung colonization of tumor cell in the absence of LPS pretreatment. However, LPS increased the number of colonization nodules and the weight of the lung in both WT and *Hectd3*^−/−^ mice. However, the increase of lung colonization of tumor cell induced by LPS was significantly compromised in *Hectd3*^−/−^ mice (Fig. 1f–h). Consistently, *Hectd3* KO significantly prolonged mouse survival (Fig. 1i). Similar results were observed when we used 4T1-Luc2 breast tumor cells and B16-F10 melanoma cells (Supplementary Fig. 1f–k). When we replaced inflammation factor LPS with TNFα, *Hectd3* KO also significantly inhibited lung colonization of 4T1-Luc2 breast tumor cells (Supplementary Fig. 1l–n). Taken together, we conclude that Hectd3 deficiency suppresses the inflammation-induced lung colonization of tumor cell.

### HECTD3 promotes adhesion of tumor cells to human umbilical vein endothelial cells (HUVECs) by upregulating E-selectin, ICAM-1 and VCAM-1 expression

**Fig. 2:**
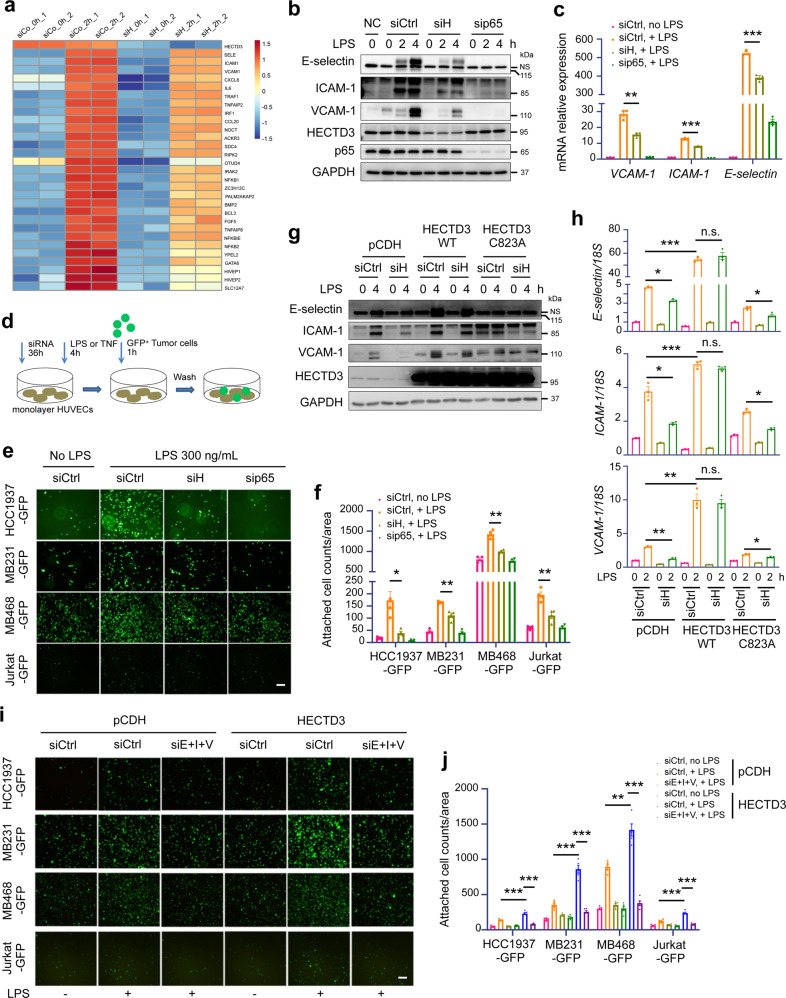
HECTD3 promotes adhesion of tumor cells to HUVECs by upregulating E-selectin, ICAM-1 and VCAM-1 expression in HUVECs. **a** RNA-sequencing analysis of gene expression in untreated and TNFα-treated control and HECTD3 knockdown HUVECs for 2 h. Heat map showing the expression of genes responsive to TNFα in untreated control HUVECs (siCo_0h), TNFα-treated control HUVECs (siCo_2h), untreated HECTD3 KD HUVECs (siH_0h) and TNFα-treated HECTD3 KD HUVECs (siH_2h). Each group has two experimental repeats. **b** Immunoblot analysis of adhesion molecules, like E-selectin, ICAM-1 and VCAM-1 in HUVECs knocking down HECTD3 or p65 using corresponding siRNA for 36 h, and stimulated with or without LPS (300 ng/mL) as indicated time. siControl (siCtrl) targeted nothing and siHECTD3 (siH) was a siRNA pool containing siHECTD3 1# and 2# here and in the following experiments. NC, negative control. NS, nonspecific band. **c** qRT-PCR analysis of adhesion molecules in HUVECs knocking down HECTD3 or p65 and stimulated with LPS (300 ng/mL) for 2 h. **d** Schematic representation of the in vitro adhesion assay. HECTD3 or p65 was knocked down in HUVECs, and cells were seeded into 6-well plates. HUVECs were treated with LPS (300 ng/ml) or TNFα for 4 h when the cells became fully confluent. Then, suspended GFP-labeled tumor cells were added and incubated for 1 h. Unattached cancer cells were washed away, and cancer cells adhered to HUVECs were quantified. **e** Representative images of the adhesion of GFP-labeled tumor cells to monolayer-cultured HUVECs transfected with the indicated siRNA and stimulated with or without LPS. **f** Bar graphs show the number of GFP-labeled tumor cells attached to monolayer-cultured HUVECs of panel **e**. **g** HUVECs stably overexpressing siRNA-resistant HECTD3, HECTD3 C823A mutant, and control (pCDH) were established. Immunoblots of these HUVECs knocked down endogenous HECTD3 or not and stimulated with LPS (300 ng/ml) for 4 h. **h** HUVECs stably overexpressing siRNA-resistant HECTD3, HECTD3 C823A mutant, and control (pCDH) were established. qRT-PCR analysis of these HUVECs knocked down endogenous HECTD3 or not and stimulated with LPS (300 ng/ml) for 2 h. **i** Representative images of the adhesion of GFP-labeled tumor cells to monolayer-cultured HUVECs in which HECTD3 was overexpressed or not. E-selectin, ICAM-1 and VCAM-1 were simultaneously knocked down by siE+I + V, a siRNA mixture of siE-selectin, siICAM-1 and siVCAM-1. **j** Quantitative data of panel **i**. Data represent 3 independent experiments for all of the above experiments. Data are presented as the mean ± SEM, and statistics were calculated using a two-tailed *t*-test for **c**, **f**, **h**, **j**. *P < 0.05; **P < 0.01; ***P < 0.001; n.s. not significant. Scale bars, 200 μm for **e**, **i**

Adhesion of cancer cells to ECs is a key step for metastasis. Systemic inflammation provoked by surgical trauma or LPS/TNFα stimulation increases the adhesion of CTCs to the vascular endothelium of distant organs by upregulating adhesion molecules in the endothelium. To determine the mechanism by which HECTD3 regulates inflammation-related metastasis, we isolated and validated primary HUVECs from the neonatal umbilical cord vein,^35^ and performed genome-wide expression analysis to profile differentially expressed genes in control and HECTD3-KD HUVECs treated with or without TNFα for 2 h. Compared with control HUVECs, 59 genes showed lower expression in HECTD3-KD HUVECs in response to TNFα. Most of them are NF-κB target genes, including adhesion molecules, such as *SELE* (E-selectin), *ICAM-1*, and *VCAM-1*, and inflammation factors, such as *IL-6* and *CXCL8* (Fig. 2a). To confirm the results of the RNA-seq, we knocked down HECTD3 in HUVECs with a siRNA pool, both protein and mRNA expression levels of adhesion molecules, including E-selectin, ICAM-1 and VCAM-1, were significantly downregulated in response to LPS (Fig. 2b, c). Similar results were observed for TNFα stimulation (Supplementary Fig. 2a, b). As a positive control, knockdown of p65/RelA abolished the induction of adhesion molecules in response to these inflammatory factors (Fig. 2b, c and Supplementary Fig. 2a, b). Knockdown of HECTD3 with two different siRNAs showed similar results (Supplementary Fig. 2c, d). Knockdown of HECTD3 in HUVEC also significantly decreased LPS- and TNFα-induced transcription of inflammation factors, such as IL-6 and CXCL8 (Data not shown). These findings suggest that HECTD3 contributes to NF-κB signaling pathway.

Next, we assessed whether HECTD3 promotes the adhesion of tumor cells to HUVECs. We treated monolayer-cultured HUVECs with LPS to induce adhesion molecule expression, added suspended GFP-labeled tumor cells to allow attachment, washed away the unattached tumor cells, and quantified tumor cells adhered to monolayer-cultured HUVECs (Fig. 2d). As expected, LPS and TNFα increased attachment of GFP-labeled breast cancer cells (HCC1937-GFP, MDA-MB-231-GFP and MDA-MB-468-GFP) and leukemia cells (Jurkat-GFP) to monolayer-cultured HUVECs (Fig. 2e, f and Supplementary Fig. 2e, f). Likewise, knockdown of HECTD3 or p65 significantly inhibited attachment of cancer cells to monolayer-cultured HUVECs (Fig. 2e, f and Supplementary Fig. 2e, f).

Subsequently, we examined whether HECTD3 functions through its E3 ligase activity. We knocked down endogenous HECTD3 and then transfected siRNA-resistant HECTD3 WT or HECTD3 C823A (a catalytically inactive mutant) constructs, which contained noncoding changes resistant to knockdown via RNAi, in HUVECs using the pCDH lentivirus overexpression system. Immunoblotting results showed that overexpression of WT HECTD3 in HUVECs significantly increased both protein and mRNA levels of E-selectin, ICAM-1 and VCAM-1 induced by LPS (Fig. 2g, h) and TNFα (Supplementary Fig. 2g, h). Interestingly, overexpressing HECTD3, but not C823A, rescued the downregulation of adhesion molecules induced by knockdown of endogenous HECTD3 with siRNA (Fig. 2g, h and Supplementary Fig. 2g, h). These results suggest that the E3 ligase activity of HECTD3 is essential for induction of adhesion molecule expression by inflammatory factors. Consistently, overexpression of WT HECTD3 but not HECTD3 C823A mutant in HUVECs significantly increased the adhesion of cancer cells to LPS-treated HUVECs (Supplementary Fig. 2i, j). Finally, we inhibited expression of E-selectin, ICAM-1 and VCAM-1 using a siRNA mixture of siE-selectin, siICAM-1 and siVCAM-1, which blocked upregulation of expression of these adhesion molecules (Supplementary Fig. 2k), as well as the increase in tumor cell adhesion (Fig. 2i, j) induced by HECTD3 overexpression in HUVECs. These results suggest that HECTD3 promotes the adhesion of tumor cells to HUVECs by upregulating the expression of E-selectin, ICAM-1 and VCAM-1 in HUVECs, which based on its E3 ligase activity.

### HECTD3 increases the transcription of adhesion molecules by stabilizing IKKα and recruiting nuclear IKKα to the promoters of adhesion molecule genes

**Fig. 3:**
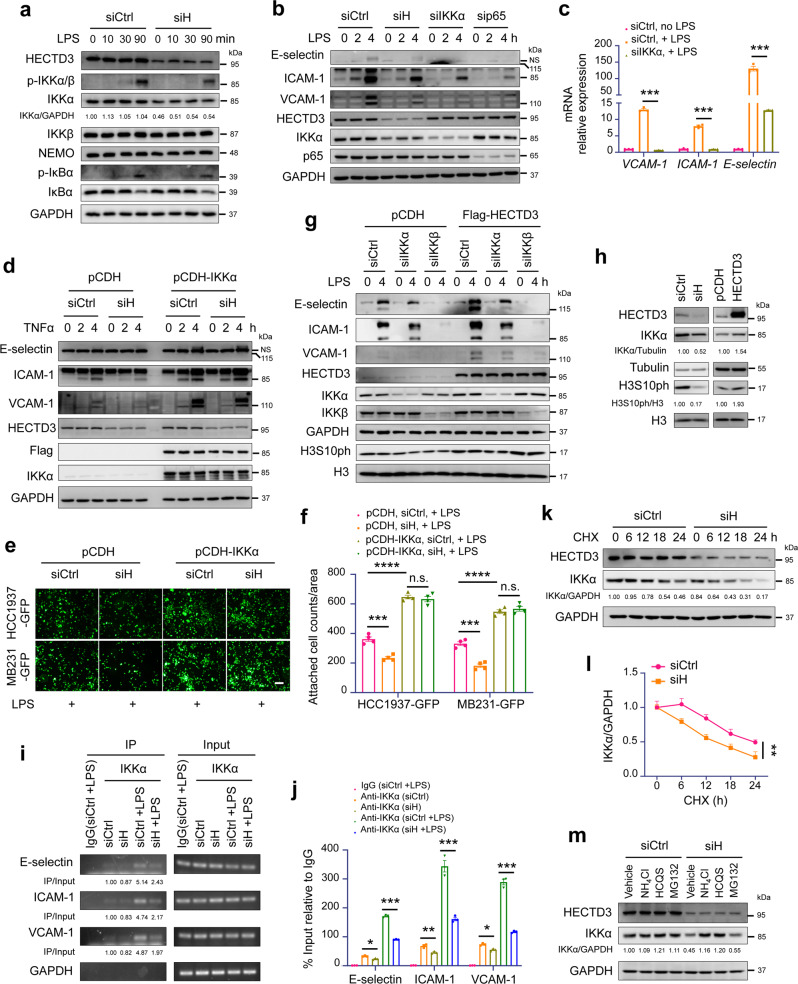
HECTD3 increases the expression of adhesion molecules by stabilizing IKKα and recruiting nuclear IKKα to adhesion molecule gene promoters. **a** Immunoblot analysis of the expression of IKKα and activation degree of the NF-κB signal pathway. HUVECs knocking down HECTD3 were stimulated with LPS (300 ng/ml) for indicated time. **b** A comparison of the expression of the adhesion molecules in HUVEC knocking down HECTD3, IKKα and p65, and stimulated with or without LPS (300 ng/mL) as indicated time. **c** qRT-PCR analysis of adhesion molecules in HUVECs knocking down IKKα and stimulated with or without LPS (300 ng/mL) for 2 h. **d** IKKα overexpression largely rescued the HECTD3 KD caused reduction of E-selectin and ICAM-1, and partially rescue the reduction of VCAM-1 in HUVECs. HUVECs were treated with TNFα (10 ng/ml) for indicated time and different proteins were detected by WB. **e** IKKα overexpression largely rescued HECTD3 KD caused reduction of adhesion phenotype. Representative images of the adhesion of GFP-labeled tumor cells to monolayer-cultured HUVECs are shown. Scale bar, 200 μm. **f** Bar graphs show the number of GFP-labeled tumor cells attached to monolayer-cultured HUVECs from panel **e**. **g** IKKα and IKKβ was transiently knocked down in HECTD3-overexpressing HUVECs stimulated with LPS (300 ng/ml) and HECTD3 overexpression-induced increases of adhesion molecule expression were blocked when IKKα or IKKβ was depleted. **h** HECTD3 positively regulated IKKα and H3S10ph levels in HUVECs. Left: HECTD3 was knocked. Right: HECTD3 was stably overexpressed in HUVECs. **i** Chromatin immunoprecipitation (ChIP) assays were performed using an anti-IKKα antibody in HUVECs transfected with siControl or siHECTD3 and stimulated with LPS (300 ng/mL) for 1 h. **j** qPCR results of the samples in panel **i**. **k** HECTD3 knockdown by siRNA decreased IKKα protein stability in HUVECs. The cells were incubated with 50 μg/ml CHX for the indicated times and were collected for immunoblotting. Tubulin was used as the internal control. The band intensity of IKKα at each time point was quantified using ImageJ. The experiments were repeated three times, and a representative experimental result is presented. **l** Quantitative data of panel **k**. **m** HECTD3 knockdown induced IKKα protein degradation through lysosomes but not proteasomes. HUVECs were treated with lysosome inhibitors (NH_4_Cl, 10 mM and HCQS, 50 μM, overnight) or proteasome inhibitor (MG132, 20 μM for 6 h) after knocking down HECTD3. Data represent three independent experiments for all of the above experiments. Data are presented as the mean ± SEM, and statistics were calculated using two-tailed *t*-test for **c**, **f**, **i**, two-way ANOVA for **l**. *P < 0.05; **P < 0.01; ***P < 0.001

NF-κB is a known signaling pathway responsible for *E-selectin*, *ICAM-1* and *VCAM-1* gene transcriptional upregulation in the endothelium in response to inflammatory stimuli. Although LPS and TNFα activate the NF-κB pathway through different receptors and adapters, the extracellular signals converge on recruitment and activation of the IKK complex, which contains IKKα, IKKβ and IKKγ (NEMO). We suspected that HECTD3 regulates a key common component of the NF-κB pathway. We first knocked down HECTD3 in HUVECs, and then stimulated cells with LPS and TNFα, and examined the expression of the IKK complex and the activation of the NF-κB signaling pathway. We found that protein levels of total IKKα, but not IKKβ or NEMO, decreased significantly (Fig. 3a and Supplementary Fig. 3a). Interestingly, the changes in p-IKKα/β, p-IκBα, and total IκBα were only slightly inhibited by HECTD3 knockdown. Thus, we subsequently focused on IKKα.

We firstly investigated whether IKKα is essential for inducing the expression of adhesion molecules by inflammatory factors. When IKKα was knocked down, the mRNA and protein expression levels of E-selectin, ICAM-1 and VCAM-1 induced by the inflammatory factors LPS (Fig. 3b, c) and TNFα (Supplementary Fig. 3b, c) in HUVECs were significantly decreased. IKKα overexpression largely rescued the reduction of E-selectin and ICAM-1, and partially rescued the reduction of VCAM-1 caused by HECTD3 KD (Fig. 3d). IKKα overexpression promoted the attachment of cancer cells to monolayer-cultured HUVECs and largely rescued the reduction of adhesion phenotype caused by HECTD3 KD (Fig. 3e, f). Furthermore, knockdown of IKKα blocked the increase of adhesion molecule expression induced by HECTD3 overexpression (Fig. 3g) and TNFα (Supplementary Fig. 3d). Expectedly, IKKβ depletion almost completely abolished these increases (Fig. 3g). These results showed that HECTD3 regulated the adhesion molecules expression and adhesion phenotype through IKKα.

Since HECTD3 knockdown decreased total IKKα protein levels but did not affect IκBα phosphorylation or degradation in response to inflammation (Fig. 3a and Supplementary Fig. 3a), we hypothesized that HECTD3 promoted adhesion molecule gene transcription in an IKK complex-independent manner. Actually, IKKα translocates into the nucleus and phosphorylates histone H3 at Ser10 and histone H3.3 at Ser31 to facilitate NF-κB-dependent transcription and it is crucial for p65 binding to the *ICAM-1* promoter’.^36^ Indeed, we demonstrated that HECTD3 knockdown significantly decreased IKKα-mediated phosphorylation of histone H3 at Ser10 (H3S10ph) and histone H3.3 at Ser31(H3.3S31ph), while HECTD3 overexpression increased H3S10ph and H3.3S31ph (Fig. 3h and Supplementary Fig. 3e, f). Consistently, we demonstrated that HECTD3 knockdown obviously decreased nuclear localization of IKKα in HUVECs under LPS stimulation by immunoblotting (Supplementary Fig. 3g) and immunofluorescence staining (Supplementary Fig. 3h). To determine whether IKKα was recruited to *E-selectin*, *ICAM-1* and *VCAM-1* gene promoters to increase transcription through epigenetic modifications, the chromatin immunoprecipitation (ChIP) assays were performed using the IKKα antibody. Nuclear IKKα does not bind to DNA sequence directly, but it can interact with p65 through CBP and is recruited to NF-κB binding chromatin to activate NF-κB target gene transcription. We designed the ChIP-PCR primers for *E-selectin*, *ICAM-1* and *VCAM-1* promoters containing a p65 binding site. As anticipated, recruitment of IKKα to these loci was obviously increased in response to LPS, while HECTD3 knockdown significantly inhibited this process (Fig. 3i, j). These results indicate that HECTD3 promotes IKKα stabilization, nuclear localization, and specific recruitment in HUVECs in response to inflammatory stimuli.

To characterize the mechanism by which HECTD3 stabilizes IKKα, we first examined *IKKα* mRNA levels after HECTD3 knockdown. There were no significant changes in *IKKα* mRNA levels (Supplementary Fig. 3i), implicating that the regulation may occur at the posttranscriptional level. Then, we used cycloheximide (CHX) to block protein synthesis and found that IKKα had a long half-life and that knockdown of HECTD3 promoted the degradation of IKKα (Fig. 3k, l). Next, we investigated the protein degradation pathway of IKKα by treating HECTD3 knockdown cells with the proteasome inhibitor MG132 or lysosome inhibitors NH_4_Cl and HCQS (hydroxychloroquine sulfate). As shown in Fig. 3m, both lysosome inhibitors, but not proteasome inhibitor, restored protein expression downregulation of IKKα induced by HECTD3 knockdown. These findings suggest that HECTD3 prevents IKKα from being degraded by lysosomes.

It’s well-known that IKKα promotes the processing of p100 precursor into p52 to activate gene transcription in noncanonical NF-κB pathway.^23^ We wondered whether HECTD3 also involved in this pathway. Expectedly, when we knocked down HECTD3 in HUVECs, the mRNA expression level of adhesion molecules, including E-selectin, ICAM-1 and VCAM-1, was downregulated in response to LTβ and BAFF (Supplementary Fig. 3j), and the protein expression level of VCAM-1 and ICAM-1 was downregulated in response to LTβ and CD40L (Supplementary Fig. 3k, l). Consistently, knockdown of HECTD3 decreased the protein level of p-p100 and inhibited the processing of p100 to p52 (Supplementary Fig. 3k).

### HECTD3 interacts with IKKα

**Fig. 4:**
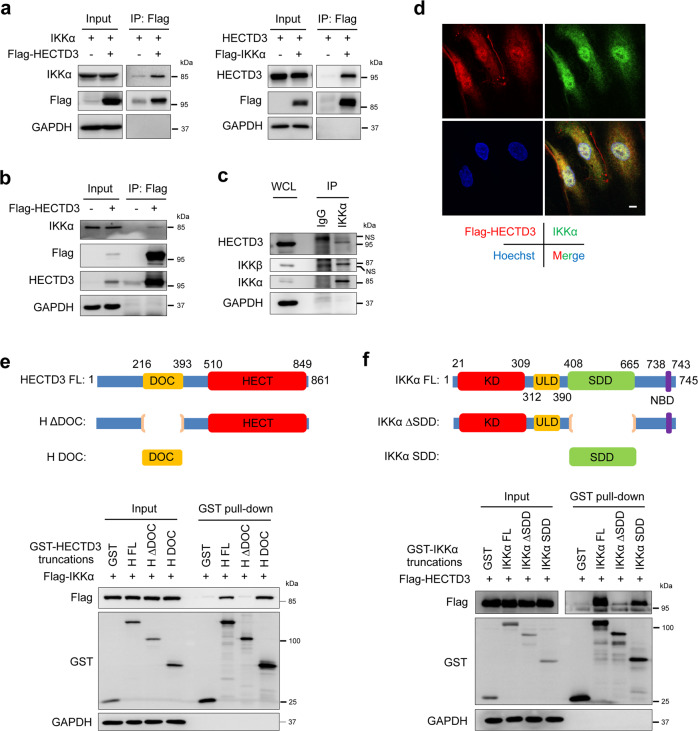
HECTD3 interacts with IKKα. **a** Exogenous HECTD3 interacts with IKKα in HEK293T cells. Flag-HECTD3 and IKKα (left) or Flag-IKKα and HECTD3 (right) plasmids were cotransfected into HEK293T cells. After Flag-tagged HECTD3 and IKKα proteins were immunoprecipitated with Flag-M2 beads, IKKα and HECTD3 were detected by immunoblotting. **b** FLAG-immunoprecipitation of FLAG-tagged HECTD3 from HUVECs. **c** Anti-IKKα antibody was used to immunoprecipitate IKKα from HUVECs. **d** Confocal microscopy images of Flag-HECTD3 and IKKα in HUVECs are shown. Scale bars, 10 μm. **e** Schematic diagram shows human HECTD3 and its truncation mutants (top). Flag-IKKα and GST-fused HECTD3 truncation mutants were coexpressed in HEK293T cells. By GST pull-down assay (bottom), GST-H DOC specifically pulled down Flag-IKKα. **f** Schematic diagram shows human IKKα and its truncation mutants (top). Flag-HECTD3 and GST-fused IKKα truncation mutants were coexpressed in HEK293T cells. By the GST pull-down assay (bottom), GST-IKKα SDD specifically pulled down Flag-HECTD3. Data represent three independent experiments for all of the above experiments

To test whether IKKα is a HECTD3 substrate, we firstly tested protein interaction between these two factors. We performed co-immunoprecipitation (co-IP) experiments and demonstrated that Flag-HECTD3 immunoprecipitated exogenous IKKα and Flag-IKKα immunoprecipitated exogenous HECTD3 in HEK293T cells (Fig. 4a). Next, we demonstrated that Flag-HECTD3 immunoprecipitated the endogenous IKKα protein in HUVECs (Fig. 4b). More importantly, endogenous IKKα and HECTD3 proteins also interact with each other, as shown using an anti-IKKα antibody in normal HUVECs (Fig. 4c). Consistently, IKKα colocalized with HECTD3 in HUVECs (Fig. 4d). However, the interaction was not increased by inflammatory factor stimulation (Supplementary Fig. 4a). Furthermore, we mapped the interaction domains of HECTD3 and IKKα. Our previous studies showed that the HECTD3 DOC domain (amino acids 216-393) is responsible for recruiting substrates, including MALT1 and Caspase-8.^26,27^ We transfected several GST-fused HECTD3 truncated mutants with Flag-IKKα in HEK293T cells and performed GST pulldown assays with glutathione sepharose beads. We found that the DOC domain also mediates the interaction between HECTD3 and IKKα (Fig. 4e). Similarly, we constructed a series of IKKα truncated mutants fused with GST and transfected them with Flag-HECTD3 in HEK293T cells, performing a GST pulldown experiment. We demonstrated that IKKα interacted with HECTD3 through its SDD domain (amino acids 408-665) (Fig. 4f and Supplementary Fig. 4b).

### HECTD3 increases IKKα protein stability, nuclear localization and kinase activity by promoting the K27- and K63-linked polyubiquitinations of IKKα at K296

**Fig. 5:**
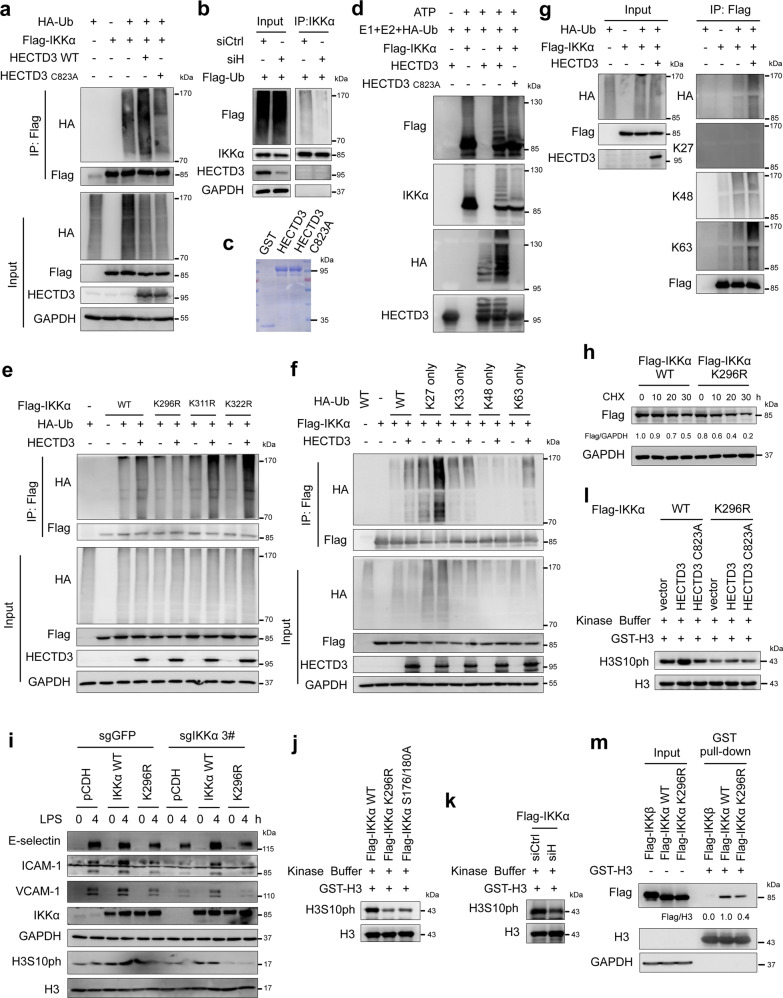
HECTD3 ubiquitinates IKKα with K27- and K63-linked polyubiquitin chains at K296 and increases IKKα protein stability and kinase activity. **a** Flag-IKKα, HA-Ub, and HECTD3 (WT) or HECTD3 C823A were coexpressed in HEK293T cells. Ubiquitinated Flag-IKKα proteins were immunoprecipitated with Flag-M2 beads and probed with anti-HA antibody. **b** Co-IP analysis of the ubiquitination of endogenous IKKα in HUVECs overexpressing stable Flag-ubiquitin (Flag-Ub). The cells were transfected with siRNA to knock down HECTD3. Anti-IKKα antibody was used for immunoprecipitation. The anti-Flag antibody was used to detect ubiquitinated IKKα. **c** Purified recombinant HECTD3 and HECTD3 C823A proteins from *E. coli* were detected by Coomassie blue staining. **d** HECTD3 ubiquitinates IKKα in vitro in an E3 ligase activity-dependent manner. ATP, HA-Ub, E1, UbcH5b, HECTD3 or HECTD3 C823A, and Flag-IKKα were mixed for ubiquitination assays. The Flag-IKKα protein was purified from HEK293 cells transfected with the plasmid encoding Flag-IKKα using Flag-M2 beads. **e** HECTD3 ubiquitinates IKKα at K296. HECTD3 failed to ubiquitinate Flag-IKKα K296R, similar to WT, K311R and K322R in HEK293T cells. **f** HECTD3 ubiquitinates IKKα with K27- and K63-linked polyubiquitin chains. WT, K27 only, and K63 only HA-Ub supported HECTD3-mediated Flag-IKKα ubiquitination. In contrast, K33 only and K48 only HA-Ub failed to do so. **g** Linkage-specific antibodies were used to validate the linkage of Flag-IKKα. **h** CHX chase assays were used to analyze the half-lives of Flag-IKKα WT and K296R mutant in HEK293T cells. **i** IKKα ubiquitination at K296 is essential for LPS to induce adhesion molecule expression in HUVECs. IKKα was stably knocked out in HUVECs using the CRISPR/Cas9 system. Immunoblotting of adhesion molecule expression in these cells restored the expression of IKKα by lentivirus encoding Flag-IKKα WT or Flag-IKKα K296R and stimulated with or without LPS (300 ng/mL) for indicated time (0–4 h). **j** The in vitro IKKα kinase assay contains purified Flag-IKKα WT, K296R, S175/180 A, GST-H3, and ATP. Flag-IKKα proteins were purified from HEK293T cells. **k** HECTD3 knockdown decreased IKKα activity toward histone H3. HECTD3 knockdown and Flag-IKKα overexpression were performed in HEK293T cells. Flag-IKKα proteins were purified for in vitro kinase assays toward GST-H3. **l** Overexpression of HECTD3, not HECTD3 C823A, increased IKKα activity toward histone H3. Flag-IKKα and Flag-IKKα K296R proteins were purified from HEK293T cells cotransfected with plasmids encoding Flag-IKKα or Flag-IKKα K296R with HECTD3 or HECTD3 C823A. In vitro kinase assays of Flag-IKKα WT and Flag-IKKα K296R toward GST-H3 were performed. **m** Flag-IKKα K296R decreased the interaction with H3 compared to Flag-IKKα WT. Cell lysates of HEK293T cells expressing Flag-IKKα WT or Flag-IKKα K296R were collected and incubated with purified GST-H3 protein for 30 min on ice. The GST pull-down assay was performed using glutathione sepharose beads. Data represent three independent experiments for all of the above experiments

Next, we investigated whether HECTD3 ubiquitinates IKKα. As expected, HECTD3, but not the HECTD3 C823A inactive mutant, significantly increased polyubiquitination of IKKα in HEK293T cells (Fig. 5a). Additionally, we showed that knockdown of HECTD3 decreased endogenous IKKα polyubiquitination in HUVECs (Fig. 5b). Moreover, we performed an in vitro ubiquitination assay using purified components, including E1, E2 (UbcH5b), E3 (HECTD3 or HECTD3 C823A) (Fig. 5c), Flag-IKKα, HA-Ub, and ATP. As shown in Fig. 5d, HECTD3 dramatically increased Flag-IKKα polyubiquitination in an E3 ligase activity-dependent manner.

The IKKα protein contains 51 lysine (K) residues. To identify the lysine residues responsible for HECTD3-mediated polyubiquitination, we artificially divided IKKα into 9 regions and constructed a series of mutants termed IKKα R1-9 in which we replaced all lysine residues with arginine (R) residues. We found that HECTD3 induced polyubiquitination of IKKα (WT) and its mutants, with the exception of IKKα R4 (Supplementary Fig. 5a). These results implied that region 4 of IKKα, which includes K296, K311, and K322, contains potential ubiquitination sites. We further identified K296 as a unique ubiquitination site because HECTD3 could not increase polyubiquitination of IKKα K296R (Fig. 5e). Taken together, these findings suggest that HECTD3 catalyzes the polyubiquitination of IKKα at K296.

Next, we determined the linkage of IKKα polyubiquitination mediated by HECTD3. Using a series of Ub mutants (K only), we found that K27- and K63—only Ub supported HECTD3-catalyzed IKKα polyubiquitination, similar to WT Ub (Fig. 5f and Supplementary Fig. 5b). We further confirmed this result using linkage-specific anti-Ub antibodies. HECTD3-mediated IKKα polyubiquitination was recognized by specific antibodies against K27-polyUb and K63-polyUb but not K48-polyUb (Fig. 5g). Consistently, knockdown of endogenous HECTD3 specifically decreased both K27-linked and K63-linked, but not K48-linked, polyubiquitination of IKKα in HEK293T cells (Supplementary Fig. 5c). These findings suggest that HECTD3 ubiquitinates IKKα with a mixture of K27-linked and K63-linked polyubiquitin chains.

To test the consequence of IKKα ubiquitination by HECTD3, we first compared the IKKα protein stabilities of K296R and WT in HEK293T cells. Compared to WT, K296R exhibited a shorter protein half-life, as measured by CHX chase experiments (Fig. 5h). Unlike WT IKKα, K296R failed to promote expression of E-selectin, ICAM-1 and VCAM-1 in response to TNFα (Supplementary Fig. 5d). Additionally, overexpression of WT IKKα rescued HECTD3 knockdown-induced downregulation of adhesion molecule expression in HUVECs under TNFα stimulation, but K296R failed to do so (Supplementary Fig. 5d). To eliminate the impact of endogenous IKKα, we generated *IKKα* KO HUVECs using CRISPR/Cas9 technology. IKKα depletion inhibited but did not abolish the expression levels of H3S10ph and E-selectin, ICAM-1 and VCAM-1 in response to LPS and TNFα stimulation (Fig. 5i and Supplementary Fig. 5e). As expected, WT IKKα, but not IKKα K296R, overexpression in *IKKα* KO HUVECs rescued the expression levels of H3S10ph and the three adhesion molecules (Fig. 5i and Supplementary Fig. 5e) and recovered the adhesion to cancer cells under inflammatory stimuli (Supplementary Fig. 5f, g). These results showed that HECTD3 promoted the expression of adhesion molecules and cancer cell adhesion through the ubiquitination of IKKα at K296.

To test whether HECTD3-mediated IKKα ubiquitination promotes IKKα nuclear translocation, we compared the subcellular distribution of IKKα WT and IKKα K296R in HUVECs treated with TNFα by collecting cytoplasmic and nuclear fractions for immunoblotting. TNFα stimulation increased nuclear localization of IKKα WT, but not K296R, in HUVECs (Supplementary Fig. 5h). This result implied that the polyubiquitination of IKKα mediated by HECTD3 is required for nuclear localization of IKKα under inflammatory conditions.

IKKα is a kinase for histone H3 and other substrates, and it is reasonable to deduce that HECTD3-mediated IKKα ubiquitination may promote its kinase activity. We next performed in vitro kinase assays to measure the kinase activities of IKKα WT, K296R, and S176/180 A proteins purified from HEK293T cells. IKKα S176/180 A is a well-known kinase dead mutant. Given that IKKα phosphorylates histone H3 at Ser10^23,24^ and IκBα at Ser32 and Ser36,^37^ we purified GST-fused histone H3 and IκBα from *E. coli* as substrates of IKKα. Notably, WT IKKα robustly phosphorylated GST-H3, while IKKα K296R and IKKα S176/180 A showed only weak kinase activities toward GST-H3 (Fig. 5j). Consistently, knockdown of endogenous HECTD3 in HEK293T cells decreased the kinase activity of IKKα (Fig. 5k), and overexpression of HECTD3, but not HECTD3 C823A, in HEK293T cells significantly increased the kinase activity of WT IKKα but not IKKα-K296R (Fig. 5l). Furthermore, similar results were obtained using GST-IκBα as the substrate in the kinase assay (Supplementary Fig. 5i, j). These results suggest that IKKα ubiquitination mediated by HECTD3 promotes IKKα kinase activity.

Dimerization is necessary for IKKα activation.^22^ Therefore, we tested whether HECTD3-mediated IKKα ubiquitination promotes its dimerization using co-IP experiments in HEK293T cells cotransfected with GST-IKKα and Flag-IKKα (WT, K296R, S176/180 A) or IKKβ. We found that the K296R mutation did not affect dimerization of IKKα (Supplementary Fig. 5k). Likewise, K296R mutation also did not influence the protein-protein interaction between HECTD3 and IKKα (Supplementary Fig. 5l). Interestingly, we found that purified GST-H3, but not GST-IκBα, pulled down more Flag-IKKα than Flag-IKKα K296R in HEK293T cell lysates (Fig. 5m and Supplementary Fig. 5m). Therefore, it is plausible that HECTD3-mediated IKKα ubiquitination increases the interaction between IKKα and histone H3 so that IKKα can efficiently phosphorylate it. However, this mechanism did not hold up for IκBα.

In order to determine whether the ubiquitination of IKKα by HECTD3 promotes its recruitment to the promoters of adhesion molecules, we knocked out the endogenous IKKα in HUVECs and restored Flag-IKKα WT or K296R mutant (Supplementary Fig. 5n). ChIP assays showed that WT, but not K296R, could be efficiently recruited to E-selectin, ICAM-1 and VCAM-1 promoters under treatment of LPS (Supplementary Fig. 5o). To test whether IKKα binds to the promoter of adhesion molecules through p65, we knocked down p65 in HUVECs and performed ChIP assays with IKKα antibody. The result showed that p65 KD significantly inhibited the recruitment of IKKα to the promoters of adhesion molecules under treatment of LPS (Supplementary Fig. 5p).

The recruitment of IKKα should promote phosphorylation of H3 at Ser10 at the promoters of adhesion molecules, thus it should promote chromatin open. In order to test this, we performed ChIP assays with H3S10ph antibody and found that HECTD3 KD in HUVECs decreased the H3S10ph level in the promoters of E-selectin, ICAM-1 and VCAM-1 under treatment of LPS (Supplementary Fig. 5q). Consistently, ChIP assays with H3K4me3 and H3K27ac antibodies showed that K296R could not efficiently promote the chromatin open (Supplementary Fig. 5r).

### Hectd3 promotes adhesion of tumor cells in the lung under inflammatory conditions

**Fig. 6:**
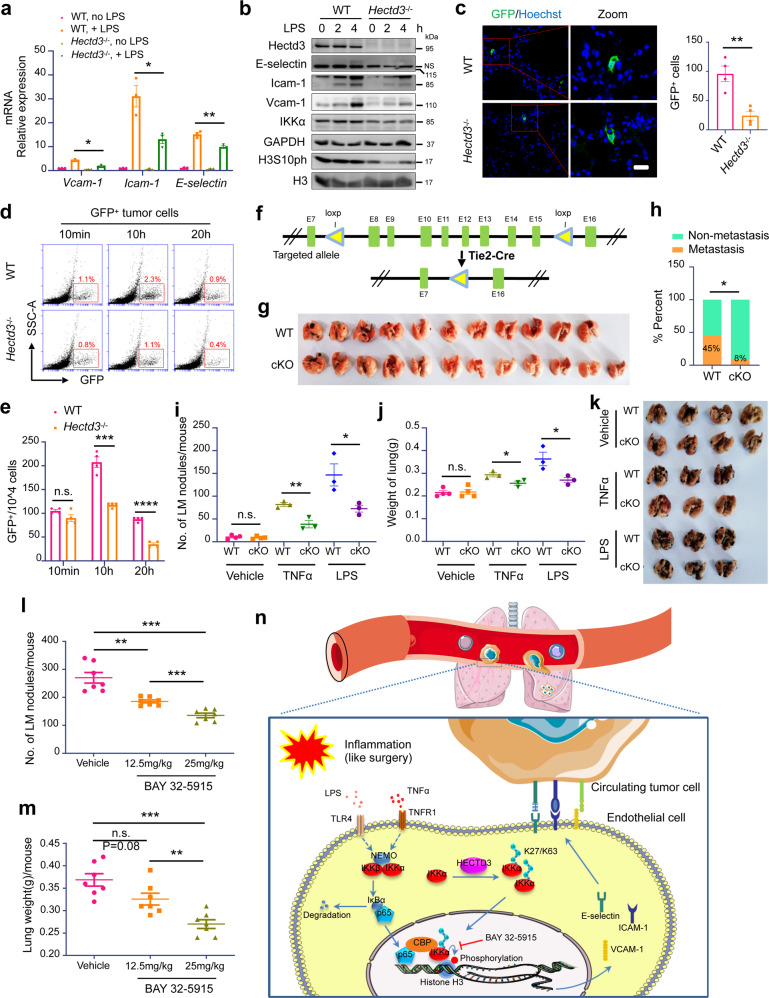
Hectd3 promotes lung colonization of tumor cells under inflammatory conditions. **a** qRT-PCR analysis of adhesion molecules in WT and *Hectd3* KO mECs stimulated with LPS (500 ng/ml) for 2 h. **b** A comparison of the expression of the adhesion molecules, IKKα and H3S10ph in mECs stimulated with or without LPS (500 ng/mL) as indicated time. **c** Representative frozen immunofluorescence images of GFP^+^ tumor cells in lungs of mice performed the tumor cell colonization assay in vivo and bar graph showing the number of GFP^+^ tumor cells in lungs of WT and *Hectd3*^−/−^ mice (right). Counting rules: Eighty sections were randomly cut from each sample (the entire lung) and then scanned by a FluoView FV1000 confocal microscope after fluorescence staining. The total number of GFP-positive tumor cells in 80 sections was recorded. **d** The GFP^+^ tumor cells (5 × 10^6^ cells per mouse) were injected through the tail vein into WT or *Hectd3*^−/−^ mice pretreated with LPS (1 mg/kg) stimulation for 5 h. At 10 min, 10 h or 20 h after tumor cell injection, the mice were sacrificed and perfused with PBS and the whole lungs were digested to cell suspension to analyze GFP^+^ tumor cell colonization in the lung by FCM. **e** The rate of GFP^+^ tumor cell in the lung of panel **d**. **f** Schematic representation of the targeted allele and the conditional allele of Hectd3 KO. **g** B16-F10 cells were injected subcutaneously into Tie2-Cre^+^;Hectd3^wt^ (*n* = 11) and Tie2-Cre^+^;Hectd3^fl/fl^ (*n* = 12) C57BL/6 mice. Twelve days after transplantation, primary tumors were removed. The mice were sacrificed at day 45 and the incidence of lung metastasis was record. Representative lung metastasis nodule image. **h** The incidence of lung metastasis of panel **g**. **i** Tie2-Cre^+^;Hectd3^wt^ and Tie2-Cre^+^;Hectd3^fl/fl^ mice were intravenously injected with vehicle, TNFα (200 μg/kg) or LPS (1 mg/kg). 5 h later, B16-F10 cells were injected through the tail vein (2 × 10^5^ cells per mouse). The mice were sacrificed 20 days after injection of tumor cells. The graph shows the number of pulmonary metastasis nodules in each group of mice. **j** The weight of the whole lung with metastatic nodules in each group of mice from panel **i**. **k** Representative lung metastasis nodule images of the lungs in different groups of mice from panel **i**. **l** 4T1-Luc2 cells were injected by tail vein into BALB/c mice (1 × 10^5^ per mouse) that were pretreated with vehicle, BAY 32-5915 (12.5 mg/kg) or BAY 32-5915 (25mk/kg) for 24 h and LPS (1 mg/kg) for 5 h by intravenous injection. The mice were sacrificed 20 days after the injection of tumor cells. The number of mouse pulmonary metastasis nodules in the three groups is shown. **m** Analysis of the weight of whole lung with metastasis nodules in the three groups from **l**. **n** The hypothetical working model. Inflammatory factors, including LPS and TNFα, activate the NF-κB pathway and promote p65 nuclear translocation and transcription of adhesion molecules, including E-selectin, ICAM-1 and VCAM-1, in HUVECs. HECTD3 ubiquitinates IKKα with K27/K63-linked polyubiquitin chains at K269 to increase IKKα protein stability, kinase activity, and recruitment to NF-κB-responsive gene promoters, where IKKα phosphorylates histone H3 at Ser10 to increase the transcription of adhesion molecules. These adhesion molecules on the EC plasma membrane promote the adhesion of tumor cells to the endothelium, leading to extravasation, colonization and metastasis. IKKα and HECTD3-specific inhibitors may prevent cancer metastasis. The Figure was partly generated using Servier Medical Art, provided by Servier, licensed under a Creative Commons Attribution 3.0 unported license (http://creativecommons.org/licenses/by/3.0/). Data are presented as the mean ± SEM, and statistics were calculated using two-tailed *t*-test for **a**, **c**, **e**, Chi-square test for **h**, two-way ANOVA for **i**, **j**, **l**, **m**. *P < 0.05; **P < 0.01; ***P < 0.001; n.s., not significant. Scale bars, 100 μm for **c**

To investigate whether Hectd3 functions similarly in mouse vascular endothelial cells, we purified mouse pulmonary vascular endothelial cells (mECs) using Dynabeads coated with anti-CD31 antibody and cultured them. *Hectd3* KO inhibited the induction of E-selectin, Icam-1 and Vcam-1 in mECs in response to LPS and TNFα stimuli, as examined by qRT-PCR (Fig. 6a and Supplementary Fig. 6a) and immunoblotting (Fig. 6b and Supplementary Fig. 6b). Consistently, *Hectd3* KO decreased the protein levels of IKKα and H3S10ph in mECs (Fig. 6a and Supplementary Fig. 6a). Immunofluorescence analysis showed that *Hectd3*^−/−^ indeed inhibited LPS-induced E-selectin protein expression in the mouse pulmonary CD31^+^ vascular endothelium (Supplementary Fig. 6c). We also examined the expression level changes of IKKα, E-selectin, VCAM-1, and ICAM-1 protein in the endothelial cells of the mice before and after surgical resection of the primary tumors by immunofluorescence staining. *Hectd3*^−/−^ dramatically decreased IKKα nuclear translocation after surgery (Supplementary Fig. 6d) and slightly inhibited IKKα expression (Supplementary Fig. 6h) in CD31^+^ endothelia. The expression levels of E-selection, VCAM-1 and ICAM-1 were significantly induced by surgery and *Hectd3*^−/−^ could significantly inhibit the induction (Supplementary Fig. 6e–g, i–k). These results suggest that Hectd3 activates IKKα and promotes adhesion molecule expression in mECs under inflammatory conditions.

As shown at the beginning of this study, *Hectd3*^−/−^ mice exhibited significantly decreased lung colonization of tumor cells which were intravenously injected into mice treated with LPS in advance. We next examined whether Hectd3 deficiency suppresses metastasis by downregulating adhesion molecule expression to inhibit tumor cell colonization in the lung. To address this question, we conducted in vivo tumor cell adhesion assays (Supplementary Fig. 6l) by injecting GFP-labeled PyMT-induced mouse breast tumor cells into WT or *Hectd3*^−/−^ mice through the tail vein. Mice were treated with LPS stimulation for 5 h before tumor cell injection and sacrificed and perfused with PBS 20 h after tumor cell injection. Immunofluorescence was performed to detect GFP-positive tumor cells in the lung. As a result, the number of tumor cells infiltrated into WT mouse lung tissues is more than that into *Hectd3*^−/−^ mouse lung tissues (Fig. 6c). We confirmed this result by digesting the lung tissues of WT and *Hectd3*^−/−^ mice and performing flow cytometry analyses. We detected the number of GFP-labeled tumor cells in the lungs in a time course experiment and found that, at 10 min after injection, the number of GFP-positive tumor cells in the lungs of *Hectd3*^−/−^ mice was almost equal to that in WT mice, but significantly decreased at 10 h and 20 h after injection (Fig. 6d, e). To test whether the increased lung colonization of GFP-positive tumor cells was caused by differential survival of tumor cells in WT and *Hectd3*^−/−^ mice under LPS stimulation, we detected the apoptosis of GFP-labeled tumor cells in the lung and found that, at 10 min, 10 h, and 20 h after intravenous injection of GFP^+^ tumor cells, the percentage of apoptotic GFP^+^ tumor cells had no significant difference between *Hectd3*^−/−^ mice and WT mice pretreated with LPS (Supplementary Fig. 6m, n). These results clearly indicated that, compared to WT, Hectd3 deficiency did not affect the survival of the foreign tumor cells in the lung, but decreases lung adhesion of tumor cells under inflammatory conditions.

To further confirm that Hectd3 deficiency inhibited tumor metastasis specifically through endothelial cells, we generated C57BL/6 strain Hectd3 floxed mice (Supplementary Fig. 6o–q) and crossed these mice with Tie2-Cre mice to obtain *Tie2-Cre*^+^;*Hectd3*^fl/fl^ mice in which Hectd3 was specifically deleted in the endothelium (Fig. 6f). We detected the Hectd3 protein expression in mECs, muscle, liver, spleen and kidney of *Tie2-Cre*^+^;*Hectd3*^wt^ (WT) and *Tie2-Cre*^+^;*Hectd3*^fl/fl^ (cKO) mice. The results confirmed that mECs isolated from cKO mice lost the expression of Hectd3, but the tissues from muscle, liver, spleen and kidney of cKO mice still expressed Hectd3 protein (Supplementary Fig. 6r). We transplanted subcutaneously B16-F10 mouse melanoma cells into WT and cKO mice, surgically removed primary tumor 12 days after transplantation and examined the lungs 45 days after resection of primary tumors. Compared to WT mice, Hectd3 cKO significantly inhibited lung metastasis (Fig. 6g, h). Furthermore, we pretreated mice with LPS and TNFα for 5 h and injected B16-F10 melanoma cells into the tail vein of WT and cKO. Hectd3-specific KO in the endothelium significantly inhibited LPS- and TNFα-induced lung metastasis (Fig. 6i–k). On the other hand, we created Hectd3 conditional knockin (cKI) mice in endothelial cells. We generated loxP-Stop-loxP-*Hectd3*^KI^-GFP C57BL/6 strain mice by inserting the targeting sequence of CAG pr-loxP-Stop-loxP-*Hectd3* CDS-P2A-eGFP-WPRE-pA into the Rosa26 site using the EGE system based on CRISPR/Cas9 developed by Beijing Biocytogen Co., Ltd (Supplementary Fig. 6s–u). We crossed our loxP-Stop-loxP-*Hectd3*^KI^-GFP mice with a background of Cre recombinase expression driven by the Tie2 promoter, which allows specific deletion of the Stop sequence to overexpress Hectd3 and GFP in the endothelium (Supplementary Fig. 6v). We isolated pulmonary vascular endothelial cells from *Tie2-Cre*^+^;*Hectd3*^KI^ and *Tie2-Cre*^-^;*Hectd3*^KI^ mice and assessed the expression of Hectd3 and GFP. As expected, Hectd3 and GFP protein expression levels were robustly increased in mECs from *Tie2-Cre*^+^;*Hectd3*^KI^ mice (Supplementary Fig. 6w). Importantly, IKKα protein levels were also increased in the endothelium of *Tie2-Cre*^+^;*Hectd3*^KI^ mice (Supplementary Fig. 6w). Subsequently, we treated mice with LPS for 5 h and injected B16-F10 melanoma cells into the tail vein. Twenty days later, *Tie2-Cre*^+^;*Hectd3*^KI^ mice showed significantly increased pulmonary metastasis compared to *Tie2-Cre*^−^;*Hectd3*^KI^ control mice (Supplementary Fig. 6x–z). These animal experimental data confirm that Hectd3 promotes tumor metastasis by enhancing tumor cell colonization mediated by vascular endothelial cells in response to inflammation.

### IKKα kinase inhibitor BAY 32-5915 suppresses lung metastasis

HECTD3 promotes adhesion molecule expression by ubiquitinating IKKα, and *Hectd3* KO inhibits tumor metastasis. Currently, there are no effective HECTD3 inhibitors available, so we examined whether an IKKα inhibitor could suppress tumor metastasis. The small molecule 8-hydroxyquinoline-2-carboxylic acid (BAY 32-5915) is a reported IKKα-specific kinase inhibitor.^38^ We demonstrated that BAY 32-5915 significantly inhibited induction of H3S10ph and adhesion molecules in HUVECs in response to LPS or TNFα (Supplementary Fig. 7a, b). To our surprise, levels of p-IKKα/β and p-p65 in HUVECs treated with BAY 32-5915 were significantly increased over time under the stimulation of LPS and TNFα, while there were no significant changes in the phosphorylation or degradation of IκBα (Supplementary Fig. 7a, b). This was likely caused by a negative feedback mechanism of IKKα inhibition by BAY 32-5915, which somehow activated a compensatory pathway. Next, we pretreated HUVECs with different concentrations of BAY 32-5915 for 12 h and then added either LPS or TNFα for 4 h. BAY 11-7082, a classic inhibitor of the NF-κB pathway, was used as a positive control. Results showed that BAY 32-5915 inhibited expression of H3S10ph, E-selectin, ICAM-1, and VCAM-1 in HUVECs in a concentration-dependent manner (Supplementary Fig. 7c). Consistently, immunofluorescence analysis showed that BAY 32-5915 actually inhibited LPS-induced E-selectin expression in the lung CD31^+^ vascular endothelium of mouse (Supplementary Fig. 7d).

To test whether BAY 32-5915 could inhibit tumor metastasis in vivo, we pretreated BALB/c mice with BAY 32-5915 (12.5 or 25 mg/kg) for 24 h and LPS (1 mg/kg) for 5 h by intravenous injection, and then injected 4T1-Luc2 cells through the tail vein. We found that the number of pulmonary metastatic nodules and the lung weight gradually decreased with increasing BAY 32-5915 concentrations (Fig. 6l, m). Pretreatment with BAY 32-5915 prolonged the survival of mice with tumor metastasis (Supplementary Fig. 7e). These results indicate that the IKKα kinase inhibitor BAY 32-5915 suppresses tumor lung metastasis induced by inflammation.

## Discussion

Cancer metastasis is a multistep process by which tumor cells disseminate from their primary site, circulate in the vessels, and eventually form secondary tumors at a distant site. Most CTCs expire in the bloodstream due to shear stress and attack of the immune system, but a small proportion of tumor cells infiltrate distant organs and survive. Surgery, including biopsy, is a double-edged sword that removes the primary tumor but induces tumor-dormancy escape and subsequent metastatic outgrowth by impairing tumor-specific immunity^39^ or by producing a transient immunosuppressive state associated with wound healing.^40^ In other words, surgery triggers abundant detachment of tumor cells readily drilling into the vasculature^3,4^ and induces systemic inflammation that assists adhesion of tumor cells to distant ECs.^5,41^ A retrospective study examining the incidence of cancer recurrence in patients with lung cancer surgery showed that perioperative treatment with ANP, which inhibits the expression of E-selectin on ECs, improved relapse-free survival after surgery compared to surgery alone.^10^ Another retrospective analysis of tumor recurrence in patients undergoing breast cancer surgery revealed that preoperative treatment with ketorolac was related to a significant decrease in recurrence and mortality after surgery.^42^ Surprisingly, preoperative stimulation with ketoralac and resolvins (RvD2, RvD3, or RvD4) for resolution of inflammation dramatically reduced lung metastasis aroused by primary tumor removal in multiple mouse models.^33^ Surgery also increases the recruitment of myeloid-derived suppressor cells (MDSCs) into the lung to form premetastatic niches, and blockade of this recruitment with 5-azacytidine and entinostat effectively inhibits lung metastasis in a mouse model.^43^ Therefore, it is important to identify additional therapeutic targets and drugs to prevent cancer metastasis caused by inflammation.

In this study, we provided evidence to support the notion that HECTD3 promotes tumor cell adhesion to ECs and metastasis by ubiquitinating IKKα in response to inflammation. First, we demonstrated that *Hectd3* KO inhibited distant tumor relapse in spontaneous metastasis models in response to surgery or systemic inflammation. Second, HECTD3 depletion in HUVECs and mouse ECs blocked inflammation-induced adhesion molecule expression and tumor cell adhesion to ECs in vitro and in vivo. In addition, Hectd3 conditional depletion in mECs inhibited tumor cell lung colonization in vivo, while Hectd3-specific overexpression in mECs increased that. Moreover, we characterized the molecular mechanism by which HECTD3 promotes metastasis. HECTD3 ubiquitinates IKKα with K63- and K27-linked polyubiquitin chains at K296, which prevents IKKα degradation by lysosomes and increases nuclear IKKα kinase activity. Activated IKKα is recruited to the promoters of adhesion molecules and phosphorylates histone H3 to facilitate transcription. Finally, we showed that an IKKα kinase inhibitor significantly suppressed inflammation-induced adhesion molecule expression and cancer metastasis in vivo. Taken together, the HECTD3-IKKα axis may serve as an effective prevention target for inflammation-induced cancer metastasis (Fig. 6n). However, Hectd3 conditional depletion in endothelium efficiently inhibited metastasis, but these results did not rule out that Hectd3 promotes metastasis through other mechanisms. In our previous study,^30^ Hectd3 deletion decreased type I interferon production in macrophages. Macrophages have an important regulatory role in metastasis, like induction of cancer cell EMT and promotion of premetastatic niche formation.^44^ Therefore, it is remained to explore whether Hectd3 also promotes metastasis through macrophages.

It has long been recognized that metastasis can be enhanced by acute or chronic inflammation, such as in response to IL-1β or LPS, which induces endothelial adhesion molecules that facilitate adhesion of cancer cells to ECs. Adhesion molecules, such as E-selectin, ICAM-1 and VCAM-1, have fundamental functions in leukocytes and hemostasis by mediating the rolling of leukocytes on activated ECs and transmigration through endothelial cell junctions. Unfortunately, tumor cells can utilize the same route to achieve distant metastasis, especially when the vascular endothelium undergoes short inflammatory stimulation, such as in response to surgery. In the bloodstream, CTCs also aberrantly express adhesion molecules to interact with platelets and immune cells, such as neutrophils, monocytes, and macrophages, as well as endothelial cells.^5^ This provides a potential approach to suppress metastasis by interrupting adhesive interactions. *E-selectin*^−/−^ mice show bone metastasis blockade.^9^ Ang2 (angiopoietin-2) increases ICAM-1 expression in ECs so that anti-Ang2 therapy limits the outgrowth of micrometastases.^32^ Anti-VCAM-1 or anti-integrin α4 mAbs also dramatically reduced bone metastasis in breast cancer.^13^ Here, we showed that HECTD3 and IKKα control inflammatory adhesion molecule expression and that inhibition of HECTD3 genetically or IKKα pharmacologically suppresses metastasis induced by acute inflammation. It is warranted to develop small molecule inhibitors for HECTD3 and IKKα for tumor metastasis prevention, especially in patients who undergo surgical removal of the primary tumor. To date, a number of small molecules like ACHP, BMS-345541, selenium-based compounds and heterocyclic adamantyl arotinoids, show inhibition to IKKα activity, but some of them also inhibit IKKβ. High selectivity remains an essential issue for IKKα inhibitor screening. E3 ligases are promising potential therapeutic targets due to their high substrate specificity. As an E3 ligase, HECTD3 can autoubiquitinate and ubiquitinate substrates in vitro, which is a good experimental basis for inhibitor screening of HECTD3.

IKKα is dispensable for IκBα phosphorylation and degradation but remains essential for NF-κB-dependent transcription because of its nuclear kinase activity. In the nucleus, IKKα is recruited to the NF-κB transcription complex to phosphorylate multiple substrates, such as CBP at Ser1382/1386, p65 at Ser536, and SMRT at Ser2410, to promote NF-κB-dependent gene transcription. Yumi Yamanoto^23^ and Vasiliki Anest^24^ independently found that nuclear IKKα was recruited to NF-κB binding chromatin and phosphorylates histone H3 at Ser10 to activate NF-κB target gene transcription after stimulation. It is well-known that H3S10ph plays a crucial role in activation of transcription. H3S10ph at promoters may lead to chromatin remodeling by recruiting 14-3-3 proteins, MSK1, and BRG1, the ATPase subunit of the SWI/SNF remodeler, to promote transcription.^45^ H3S10ph also ejects heterochromatin factors, such as HP1, HDAC1, 2 and 3,^46^ or prevents deposition of H3K9me2 associated with transcriptional repression to facilitate gene expression.^47^ Additionally, A recent study showed that IKKα phosphorylated histone variant H3.3 at S31, which deposited to the gene body, to amplify LPS-induced transcriptional elongation.^48^ All these clues indicate that IKKα may promote gene transcription initiation and elongation through H3S10ph and H3.3S31ph, respectively. Herein, we show that the ubiquitination of IKKα mediated by HECTD3 increased its kinase activity toward H3. Whether there is crosstalk between S10ph and S31ph remains unknown. Additionally, nuclear IKKα has been shown to regulate DNA damage response, radioresistance, apoptosis, and cell cycle. Whether HECTD3 regulates other functions of IKKα remains to be investigated.

Although it has been reported that IKKα ubiquitination promotes its nuclear translocation in hepatoma cells, its E3 ligase and modification details have not been fully elucidated. For the first time, we identified HECTD3 as an IKKα E3 ligase that promotes K63- and K27-linked polyubiquitination at K296. This ubiquitination modification promotes IKKα protein stability, nuclear localization and kinase activity. Furthermore, we found that blocking ubiquitination of IKKα inhibited the interaction of IKKα with the target protein histone H3 but not IκBα. However, how ubiquitination promotes the kinase activity of IKKα needs further investigation.

In summary, our data characterize the function of the HECTD3-IKKα axis in the adhesion of tumor cells to the endothelium through the NF-κB signaling pathway, which provides a potential strategy for tumor hematogenous metastasis prevention and treatment.

## Materials and methods

### Mouse strains

*Hectd3*^−/−^ mice were generated by Taconic Farms, Inc (TF2706, TACONIC KNOCKOUT REPOSITORY), with an 129/SvEv and C57BL/6 Chimeric background as mentioned in our previous study.^30^ Chimeric offspring were backcrossed to FVB genetic background for eleven generations for analysis of lung metastasis after tail vein injection of PyMT lentivirus-induced breast tumor cells, and lung and heart metastasis after removal of the orthotopic allograft PyMT lentivirus-induced breast tumor. Chimeric offspring were backcrossed to BALB/c genetic background for nine generations or C57BL/6 genetic background for eight generations to generate *Hectd3*^−/−^ mice and *Hectd3*^+/+^ mice for the similar tumor metastasis experiments using 4T1-Luc2 breast cancer cells or B16-F10 melanoma cells. The loxP-*Hectd3*-loxP C57BL/6 strain mice (project number: EGE-SSH-021-B) were generated by using the EGE system based on CRISPR/Cas9 developed by Beijing Biocytogen Co., Ltd, which were crossed with Tie2-Cre mice to generate mice with Hectd3-specific deficiency in the endothelium. These conditional KO (cKO) mice were confirmed for analysis of lung metastasis after tail vein injection of B16-F10 melanoma cells. The loxP-Stop-loxP-*Hectd3*^KI^-GFP C57BL/6 strain mice (project number: EGE-ZLY-004 KI) were generated by inserting the targeting sequence of CAG Pr-loxP-Stop-loxP-*Hectd3* CDS-P2A-eGFP-WPRE-pA at Rosa26 site by using the EGE system based on CRISPR/Cas9 developed by Beijing Biocytogen Co., Ltd. The Stop sequence is flanked by two loxP sites. Followed the second loxP site is a *Hectd3* CDS-P2A-eGFP-WPRE-pA expression element. The targeted allele was driven by chicken β-actin promoter and inserted into the Rosa26 allele in C57BL/6 background. The loxP-Stop-loxP-*Hectd3*^KI^-GFP mice were crossed with Tie2-Cre to generate the conditional knockin (cKI) mice, which allows specific overexpression of Hectd3 and GFP in the endothelium. These cKI mice were confirmed for analysis of lung metastasis after tail vein injection of B16-F10 melanoma cells. All littermate used for tumor analysis above were virgin females. All mice were kept in specific pathogen-free (SPF) conditions at the Animal Resource Center of Kunming Institute of Zoology, Chinese Academy of Sciences. All animal experiments were conducted in accordance with the guidelines and were approved by the Kunming Institute of Zoology, Chinese Academy of Sciences Animal Care and Use Committee.

### Intraductal injection of PyMT lentivirus to induce breast tumor

Wild type FVB female mice were anesthetized, mammary ducts were exposed by cutting nipple ends, and a 50 μl microsyringe was used to inject 10 μl viral concentrate of lentivirus overexpressing PyMT and GFP (FUCGW-PyMT-GFP lentivirus) into the ductal lumen of glands #4. Two weeks later, the PyMT-induced breast tumors were examined by palpation of mammary glands if the injection was successful. Resected the tumor and digested it to single-cell suspension using collagenase type III (Worthington-biochem, NJ, USA) and hyaluronidase (Sigma-Aldrich, MO, USA), which was applied to tail vein injection or orthotopic allograft transplantation.

### Spontaneous metastasis assay

WT and *Hectd3*^−/−^ mice with FVB or BALB/c genetic background received mammary fat pad transplantation of 1 × 10^5^ PyMT-induced breast tumor cells or 4T1-Luc2 cells suspended in 75 μl mixture of PBS and Matrigel (1:1; BD Biosciences, CA, USA). Twenty days (PyMT-induced tumor) or 12 days after transplantation (4T1-Luc2 tumor), surgical resection was operated to remove the tumors. In PyMT-induced tumor cell experiments, the lung and heart metastasis were record when mice being natural mortality or sacrificed 2 months after removal of tumor. In 4T1-Luc2 experiment, bioluminescence imaging 4T1-Luc2 tumor burden on day 1 before the tumor resection and tumor metastatic burden was monitored weekly by IVIS imaging after tumor removal.

### Immunoblotting analysis and antibodies

Protein samples were separated by 11% SDS-PAGE and followed by electrophoretic transfer onto PVDF membranes and blocked with 5% non-fat milk and further incubated overnight in primary antibody (dilute 1:500–5000 in 3% BSA) at 4 °C as described previously.^30^ The following primary antibodies were used: anti-E-selectin (sc-137054), anti-ICAM-1 (sc-8439), anti-VCAM-1 (sc-8304), anti-HA (SC-805) and anti-GAPDH (sc-25778) Antibodies were purchased form are from Santa Cruz Biotechnology, California. The anti-p65 (8242), anti-p-p65(3033), anti-IκBα (9242), anti-p-IκBα (9246), anti-p-IKKα/β (2078), anti-IKKα (2682), anti-IKKβ (2684), anti-NEMO (2685) anti-H3S10ph (3377), anti-H3 (4499), anti-Lamin B1(13435), anti-K48 Ub (8081), anti-K63 Ub (5621), anti-p-p100 (4810), anti-p100/52 (4882) and HRP-labeled anti-rabbit, anti-mouse or anti-goat secondary antibodies were purchased from Cell Signaling Technology (CST, MA, USA). The anti-GST (G7781) and anti-FLAG (F3165) antibodies were from Sigma-Aldrich. The anti-Ub (04–263) Ab was from Millipore(MA, USA). The anti-K27 Ub Ab (ab181537) was from Abcam (MA, USA). The anti-tubulin Ab (11224-1-AP) was from Proteintech (IL, USA). The anti-GFP Ab (11814460001) was from Roche (Basel, Switzerland). The anti-H3.3S31ph (39637) was from Active motif. Then anti-HECTD3 Antibody was previously described.^26^

### siRNAs

Small interfering RNA (siRNA) for human genes was synthesized by Guangzhou RiboBio Co., LTD as follows: HECTD3-specific siRNA (1#: sense, GCG GGA ACU AGG GUU GAA Utt; 2#: sense, GGU AUU UCA CCU CUU AAG Att), IKKα-specific siRNA (sense, GCA GGC UCU UUC AGG GA CAtt), IKKβ-specific siRNA (sense, CAGGUGAGCAGAUUGCCAU), p65-specific siRNA (sense, GCC CUA UCC CUU UAC GUC Att), E-selectin-specific siRNA (sense, CAA CAA UAG GCA AAA AGA Utt), ICAM-1-specific siRNA (sense, AGU CAA CAG CUA AAA CCU Utt), VCAM-1-specific siRNA (sense, GGA GUU AAU UUG AUU GGG Att). siRNA oligonucleotides were transfected in HUVEC cells with Lipofectamine 2000 (Invitrogen, CA, USA) according to the manufacturer’s instructions.

### Mouse ECs isolation and culture

Lung tissues from WT and *Hectd3*^−/−^ mice were removed aseptically, rinsed in 1× PBS, minced into ≈1 × 1 mm squares, and digested in 10 ml of collagenase A (1 mg/ml, Worthington-biochem) at 37 °C for 45 min with occasional agitation. The cellular digest was filtered through sterile 31 μm cell strainer (BD Falcon), centrifuged at 500 g for 10 min, and wash twice in DMEM with 5% FBS. The cell pellet was resuspended in 1 ml of 5% FBS-DMEM. Dynabeads (Dynal AS) coated with sheep anti-rabbit IgG were incubated in 1 ml of anti-mouse CD31 (MEC13.3) (BD bioscience) supernatant at 4 °C overnight and then washed three times with 5% FBS-DMEM; 1 ml of cell suspension was incubated with the washed beads at 4 °C for 30 min, washed three times with 5% FBS-DMEM and once FBS-free DMEM, and then digested for 5 min at 37 °C in 1 mL of trypsin/EDTA (Gibco/Invitrogen) to release the beads. The bead-free cells were centrifuged and resuspended in 10 ml of EBM-2 medium (Lonza, Basel, Switzerland).

### Cells culture and lentivirus infection

HUVEC cells were cultured in EBM-2 medium, HCC1937-GFP cells was cultured in RPMI1640 medium with 5% FBS, MDA-MB-231-GFP and Jurkat-GFP was cultured in DMEM/F12 medium with 5% FBS, MDA-MB-468-GFP was cultured in DMEM medium with 5% FBS. Human WT HECTD3 and HECTD3 C823A, WT IKKα and IKKα mutants were cloned into the lentiviral expression vector pCDH-CMV-MCS-EF1-Puro, and sgRNA sequences for IKKα (sgIKKα 3#: 5'-GGC CCT GGG AGA TGC GGG AG-3') and GFP (sgGFP: 5'-GTC GCC GTC CAG CTC GAC C-3') were cloned into lentiCRISPR-v2 vector. The viral particles were prepared by transfecting HEK293T cells with the constructed plasmids or control plasmids in combination with packaging vectors. 12 h later, media was replaced with fresh complete DMEM. Viral supernatant was harvested and passed through 0.45 µm syringe filter at 48 and 72 h after transfection. To establish stably infected cells, HUVECs were infected with lentivirus as indicated in the presence of polybrene (8 µg/ml) for 12 h and selected further in the presence of puromycin (0.2 µg/ml).

### In vitro tumor cell adhesion assay

To quantify tumor cell adhesion to HUVECs, a standardized cell adhesion assay was performed. Before coculture with tumor cells, HECTD3 or p65 were knocked down with siRNA in HUVECs seeded in 6-well plate, and treated with LPS (300 ng/mL) for 4 h. Suspended GFP-labeled tumor cells (HCC1937-GFP, MDA-MB-231-GFP, MDA-MB-468-GFP and Jurkat-GFP) (2 × 10^5^ cells per dish) ware added to the confluent monolayer-cultured HUVECs and cocultured for 1 h. The cells were washed three times with PBS gently to remove non-adherent tumor cells, then fixed with 4% (wt/vol) paraformaldehyde. The number of adhering GFP-positive cells in the fixed plate was counted by using images obtained with a fluorescence microscope (Nikon).

### In vivo tumor cell adhesion assay

Lentiviruses overexpressing PyMT and GFP were injected intraductally to induce breast tumors and tumor cells were digested into a single-cell suspension as described above. The single-cell suspension (5 × 10^6^ cells per mouse) was injected through the tail vein into WT or *Hectd3*^−/−^ mice pretreated with LPS (1 mg/kg) stimulation for 5 h. Twenty hour after tumor cell injection, the mice were sacrificed and perfused to analyze tumor cell colonization in the lung.

### RNA-sequencing analysis

Total RNA was extracted from HUVECs transfected with control siRNA or siHECTD3 treated with TNFα for 2 h or without using TRIzol reagent (Invitrogen, Carlsbad, CA, USA) to commercial RNA-seq analysis (LC-Bio Technology CO., Ltd., Hangzhou, China). Poly (A) RNA was purified from 1 μg total RNA per sample using Dynabeads Oligo (dT) 25-61005 (Thermo Fisher, CA, USA) for the final cDNA library with average insert size 300 ± 50 bp to perform the 2 × 150 bp paired-end sequencing (PE150) on an illumina Novaseq™ 6000 following the recommended protocol. Fastp software (https://github.com/OpenGene/fastp) was used to verify sequence quality and HISAT2 (https://ccb.jhu.edu/software/hisat2) was used to map reads to the reference genome of Homo sapiens GRCh38. StringTie (https://ccb.jhu.edu/software/stringtie) was used to assemble the mapped reads of each sample. Gffcompare (https://github.com/gpertea/gffcompare/) was used to reconstruct a comprehensive transcriptome. Then, we used StringTie to perform expression level for mRNAs by calculating FPKM. The significantly differential expressions were selected with fold change < 0.5 or fold change > 2 and with parametric F-test comparing nested linear models (*p*-value < 0.05) by R package edgeR (https://bioconductor.org/packages/release/bioc/html/edgeR.html).

### Immunofluorescence staining and microscopy

For IKKα and Flag immunostaining, LPS treated and untreated HUVECs were fixed in 4% paraformaldehyde for 15 min at room temperature. Cells were washed with 1×PBS, blocked in 5% BSA buffer with 0.1% saponin for 1 h. The cells were stained with anti-IKKα (CST, 2682) at 1:300 dilution or anti-Flag (Sigma-Aldrich, F7425) at 1:500 dilution, overnight at 4 °C. The cells were washed, stained with fluorescence conjugated secondary antibody for 1 h at room temperature, nuclear was staining by Hoechst (Invitrogen), and mounted using mounting medium (Vector Laboratories, H-1200). For lung tissues frozen immunofluorescence assay, anti-GFP (Abcam, ab13970), anti-CD31 (LBP, IHC-M033 and CST, 92841), anti-IKKα (CST, 2682), anti-E-selectin (BD, 740027), anti-VCAM-1 (Santa cruz, sc-8304), anti-ICAM-1 (Santa cruz, sc-8439) antibodies were used. The mouse lung tissues were fixed in 4% paraformaldehyde in PBS for 24 h, at room temperature. After fixation, the tissues were dehydrated with 15% sucrose in PBS for 24 h, at 4 °C. Tissue sections were permeabilized with 0.5% Triton X-100 for 20 min and blocked with 10% goat serum in PBS for 1 h, at room temperature. Primary antibodies were diluted in 1% of serum and incubated overnight, at 4 °C, and secondary antibodies were diluted in 1% of serum and incubated for 1 h, at room temperature. Nuclear was staining by Hoechst, and mounted using mounting medium. The cells were observed on the Olympus FluoView 1000 confocal microscope (Olympus) for image acquisition and data analysis.

### Chromatin immunoprecipitation assay

The crosslinking ChIP assay was performed using HUVECs following the manufacturer’s procedure (Abcam, Cambridge, MA, USA) with silight modifications. HUVECs were fixed with 1% formaldehyde for 10 min. Glycine (125 mM) was added to terminate the crosslinking reaction. Fixed HUVECs were collected and resuspended in cytoplasmic lysis buffer (85 mM KCl, 0.5% NP-40, 5 mM PIPES, pH 8.0) with protease inhibitors for 10 min. The pellet nuclei was collected by centrifuge and resuspended in 100 μL cytoplasmic lysis buffer with micrococcal nuclease (CST, #10011) to digest genome DNA at 37 °C for 30 min. The pellet nuclei were collected again and resuspended in nuclear lysis buffer (10 mM EDTA, 1% SDS, 50 mM Tris-Cl, pH 8.1) with protease inhibitors on ice for 10 min. Then, the pellet nuclei were broken by ultrasonic crusher for release of the DNA-protein complex. The DNA-protein complex derived from HUVECs was incubated with antibodies and Protein A/G beads at 4 °C for 10 h. The chromosomal DNA was purified and analyzed by normal or quantitative PCR. Anti-p-H3S10ph (CST, 53348), anti-H3K4me3 (Abcam, ab8580), anti-H3K27ac (Abcam, ab4729) and anti-IKKα (CST, 2682) antibodies were used for ChIP assays. Primers for ChIP as followed: (human) E-selectin: forward 5'- CGG GAA AGT TTT TGG ATG C-3', reverse 5'- GAG GGA TTG CTT CCT GTG AA -3'; ICAM-1: forward 5'- GGG GCG GGA ATT CAG AAC-3', reverse 5'- GCC ATC CAG AGA CGC ATA TT -3'; VCAM-1: forward 5'- TTG GCT GGG TGT CTG TTA AA -3', reverse 5'- TAA AGG GTC TTG TTG CAG AGG -3'.

### Co-immunoprecipitation and GST Pull-down

HECTD3 and IKKα were cloned into pCDH-CMV-MCS-EF1-puro-3×Flag or pLenti6 vector. Truncated mutants of HECTD3 and IKKα were cloned into pEBG-GST vector. Lipofectamine 2000 reagents (Invitrogen) were used for transient transfection of plasmids into HEK293T cells. For immunoprecipitation (IP), whole HEK293T cells collected 48 h post-transfection, HUVECs and HUVECs overexpressed Flag-HECTD3 were lysed in Pierce IP lysis buffer composed of 1.0% (vol/vol) NP-40, 25 mM Tris-HCl pH 7.4, 1 mM EDTA, 150 mM NaCl, 5% glycerol (vol/vol) and protease/phosphatase inhibitor cocktails (Sigma-Aldrich). After centrifugation, supernatants were collected and incubated with Flag-M2 beads (Sigma-Aldrich, F3165) or protein A/G Plus-Agarose (Santa Cruz, SC-2003) and 5 μl of the IKKα antibodies (CST, 2682) for 6 h at 4 °C, followed by washing five times with Pierce IP lysis buffer. For GST pull-down, whole HEK293T cells collected 48 h post-transfection were lysed in Pierce IP lysis buffer. GST-fused proteins were pulled down with glutathione sepharose 4B beads (GE Healthcare, Uppsala, Sweden). Immunoprecipitated or pull-down components were eluted by boiling in the 1% (wt/vol) SDS sample buffer (60 mM Tris-HCl (pH 6.8), 1% (wt/vol) SDS, 5% (vol/vol) glycerol, trace bromophenol blue and 1% (vol/vol) 2-mercaptoethanol) for 10 min. For immunoblotting analysis, immunoprecipitated samples and input lysates were separated by SDS-PAGE, followed by transferring onto PVDF membranes and detected by specific antibodies.

### Ubiquitination analysis

For polyubiquitination analysis of IKKα in HEK293T cells, the cells were transfected with plasmids expressing WT HECTD3 or inactivated mutant HECTD3 C823A, HA-ubiquitin (WT, K6 only, K11 only, K27 only, K29 only, K33 only, K48 only, K63 only, or K0), and Flag-IKKα (WT and mutants). 36 h after transfection, the cells were harvested in 200 μL SDS lysis buffer (1.5% SDS, 50 mM Tris-Cl, pH 6.8) and boiled for 15 min. 60 μL of the cell lysates was isolated as Input and the remaining was diluted with 1.2 mL cold BSA buffer (0.5% BSA, 0.5% NP-40, 180 mM NaCl, 50 mM Tris-Cl, pH 6.8) and immunoprecipitated with the Flag-M2 beads for 6 h at 4 °C with rotation. The beads were collected by centrifuge with 500 g for 2 min at 4 °C and washed four times with cold BSA buffer. The beads were boiled for 5 min with 65 μL SDS sample buffer and analyzed by immunoblotting with anti-HA to detect IKKα ubiquitination. For polyubiquitination analysis of endogenous IKKα in HUVECs, HUVECs overexpressed Flag-Ub were transfected with siRNA targeting HECTD3 for 36 h, and the cells were harvested as mentioned above and the cell lysates were immunoprecipitated with the anti-IKKα antibody and analyzed by immunoblotting with anti-Flag to detect IKKα ubiquitination. For In vitro polyubiquitination analysis of IKKα, we assembled the reaction in vitro with all purified components, including Mg-ATP (25 μM), HA-Ub (200 ng), E1 (100 ng), UbcH5b (200 ng), HECTD3 or HECTD3 C823A (500 ng) and Flag-IKKα as substrate in 40 μl ubiquitin conjugation reaction buffer. GST-fused HECTD3 and HECTD3 C823A were purified from *E. coli* BL21 (DE3) with glutathione sepharose beads, and GST was removed by digestion using 3 C protease. Flag-IKKα was purified from HEK293T cells transfected plasmid encoding Flag-IKKα using Flag-M2 beads. Other components were purchased from Boston Chem. Reactions were incubated for 1 h at 37 °C and stopped with standard SDS-PAGE loading buffer (β-mercaptoethanol containing). The samples were analyzed by immunoblotting with specific antibodies.

### Kinase assays in vitro

Flag-IKKβ, Flag-IKKα and its mutants Flag-IKKα K296R and S176/180 A were cloned into pCDH-CMV-MCS-EF1-puro-3×Flag vector. The plasmids were transfected in HEK293T cells with Lipofectamine 2000. After 36 h, cell extracts were immunoprecipitated with anti-Flag M2 beads. Histone H3 and IκBα were cloned into pGEX-6p-1 vector and transduced into DE3 (BL21) *E. coli*. GST- H3 and GST-IκBα proteins were purified with glutathione sepharose beads. Briefly, the immunoprecipitates were incubated with GST-H3 or GST-IκBα in the kinase reaction buffer which contains 20 mM HEPES at pH 7.5, 10 mM MgCl_2_, 20 mM b-glycerophosphate, 10 mM PNPP, 50 mM Na_3_VO_4_, 1 mM DTT, 20 mM ATP, and at 30 °C for 30 min. The products were subjected to SDS-PAGE and immunoblotting with anti-H3S10ph antibody or anti-p-IκBα (Ser32/36) antibody.

### Real-Time quantitative PCR

Total RNA was isolated from tissues or cells using Trizol (Invitrogen) and purified by RNeasy Mini Kit (QIAGEN), cDNA was reverse transcribed by using iScript cDNA Synthesis Kit (Bio-Rad, CA, USA). Primers were designed according to the published sequences and listed as follows: (human) E-selectin: forward 5'-CTG GCT TCG GAA ATG CTT AC-3', reverse 5'-CCA GAG ACC CGA GGA GAG TT-3'; ICAM-1: forward 5'-GAG CAC TCA AGG GGA GGT C-3', reverse 5'-CAT TAT GAC TGC GGC TGC TA-3'; VCAM-1: forward 5'-GAA CCC AAA CAA AGG CAG AG-3', reverse 5'-GGA TTT TCG GAG CAG GAA AG-3'; 18 S: forward 5'-CGC CGC TAG AGG TGA AAT TCT-3', reverse 5'-CGA ACC TCC GAC TTT CGT TCT-3'; GAPDH: forward 5'-GGT GAA GGT CGG AGT CAA CG-3', reverse 5'-TGG GTG GAA TCA TAT TGG AAC A-3'; HECTD3: forward 5'-ACC GTT CTC GTT TCA TCC AA-3', reverse 5'-GGT ACC ACC TTC AGC AGA CC-3'; IKKα: forward 5'-CAA ATG AGG AAC AGG GCA AT-3', reverse 5'-CTT CCA TAG GTT TGG GGA CA-3'; (mouse) E-selectin: forward 5'-TGA CATC GTC CTC ATT GCT C-3', reverse 5'-CAA ACG ATT GAA GGC TTT GG-3'; Icam-1: forward 5'-TAC GTG TGC CAT GCC TTT AG-3', reverse 5'-CCT TGA GTT TTA TGG CCT CCT C-3'; Vcam-1: forward 5'-ACG AGG CTG GAA TTA GCA GA-3', reverse 5'- GAC GGT GTC TCC CTC TTT GA-3'; Hectd3: forward 5'-TGA CCA GTG GTG GAA TGT A-3', reverse 5'-TGA CAT ATC TGC CAG GCT GT-3'; GFP: forward 5'-ACG ACG GCA ACT ACA AGA CC-3', reverse 5'-GTC CTC CTT GAA GTC GAT GC-3'. Real-time qPCR was performed on the 7900HT fast real-time PCRsystem (Applied Biosystems) with SYBR Green Select Master mix (Applied Biosystems). Data were normalized based on the 18 S or GAPDH expression levels.

### Statistical analysis

Data are given as mean ± SEM. Statistical analyses were performed using two-tailed *t*-test, two-way ANOVA, Log-rank test or Chi-square test. *P*-values ≤ 0.05 were considered significant.

## Supplementary information

Supplementary information

## Data Availability

The data that support the findings of this study are available from the authors upon reasonable request. The RNA-seq data are submitted at Gene Expession Omnibus (http://www.ncbi.nlm.nih.gov/geo) under record number GSE201759. These data can be accessed with the following link. https://www.ncbi.nlm.nih.gov/geo/query/acc.cgi?acc=GSE201759.
