# Supplementary material for: Targeting HECTD3-IKKα axis inhibits inflammation-related metastasis
Source: Signal Transduct Target Ther. 2022 Aug 3;7:264. doi: 10.1038/s41392-022-01057-0 (PMC9345961; doi:10.1038/s41392-022-01057-0)
Supplement: Supplementary file 1 — Supplementary information [file 41392_2022_1057_MOESM1_ESM.docx]

Supplementary Materials for

**Targeting HECTD3-IKKα axis inhibits inflammation-related metastasis**

Fubing Li^1,2 #^, Huichun Liang^1,3 #^, Hua You^2^, Ji Xiao^4^, Houjun Xia^5^, Xi Chen^1^, Maobo Huang^1^, Zhuo Cheng^1^, Chuanyu Yang^1^, Wenjing Liu^1^, Hailin Zhang^1^, Li Zeng^1^, Yingying Wu^6^, Fei Ge^6^, Zhen Li^7^, Wenhui Zhou^8^, Yi Wen^1^, Zhongmei Zhou^1^, Rong Liu^1^, Dewei Jiang^1^, Ni Xie^9^, Bin Liang^10^, Zhenzhen Liu^11 *^, Yanjie Kong^9 *^, Ceshi Chen^1 *^

Correspondence to: Ceshi Chen, Tel: 86-871-65181944, E-mail: [chenc@mail.kiz.ac.cn](about:blank), Yanjie Kong, Email: [kongyanjie26@163.com](mailto:kongyanjie26@163.com), or Zhenzhen Liu: zlyyliuzhenzhen0800@zzu.edu.cn

**This PDF file includes:**

Figures. S1 to S7

**
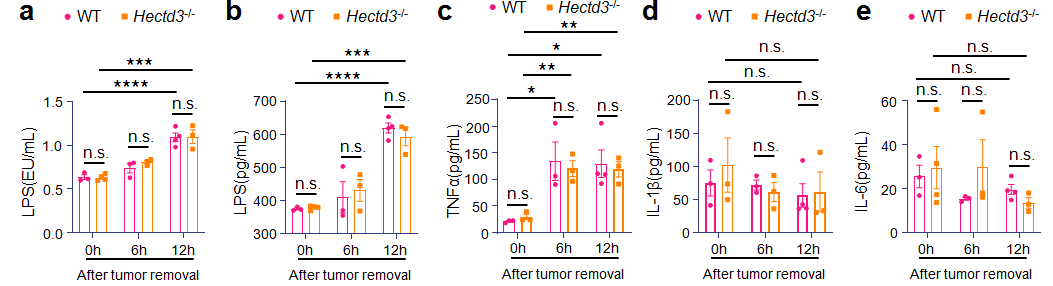

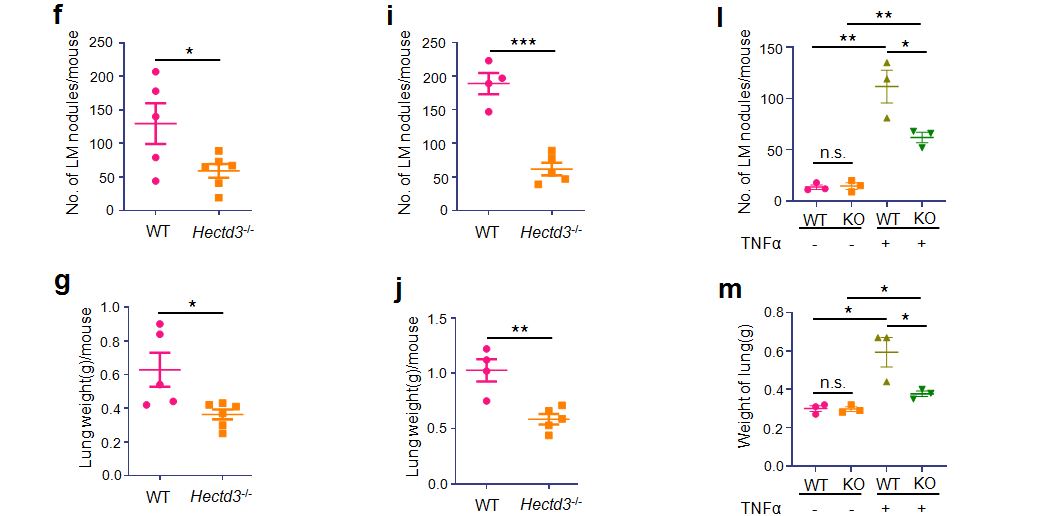

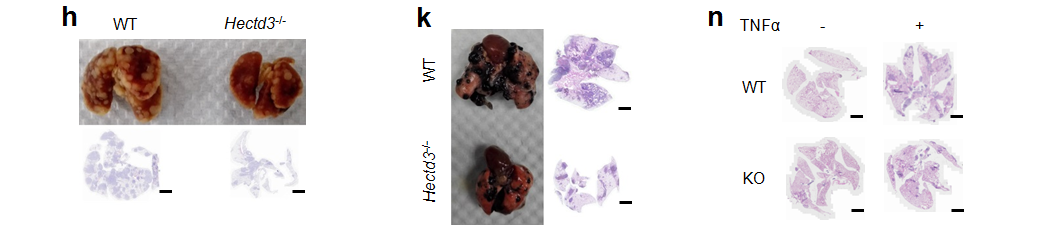
**

Figure. S1 *Hectd3* knockout inhibits tumor metastasis in different mouse models.

**a-e** 4T1-Luc2 cells were orthotopically transplanted to WT and *Hectd3*^-/-^ BALB/c mice. Twelve days after transplantation, removed the primary tumors, then collected the serum 6 h or 12 h later for testing the serum level of LPS (**a**, **b**), TNFα (**c**), IL-1β (**d**) and IL-6 (**e**), the serum from mice being normal and burdening primary tumor as control group. ELISA kits for inflammatory factors: LPS (spbio, SP14143), TNFα (4A Biotech, CME0004), IL-1β (4A Biotech, CME0015), and IL-6 (4A Biotech, CME0006); Limulus amebocyte lysate assay kit for LPS: XIAMEN BIOENDO TECHNOLOGY, EC32545S. **f** *Hectd3* KO decreased lung metastasis of 4T1-Luc2 breast tumor cells. 4T1-Luc2 cells were injected by tail vein into WT (n=5) and *Hectd3*^-/-^ (n=6) BALB/c mice (1×10^5^ per mouse) which were pretreated with LPS (1 mg/kg) intravenous injection for 5 h. The mice were sacrificed 20 days after tumor cell injection. Graph shows the number of pulmonary metastasis nodules in each group mice. **g** The weight of whole lung with 4T1-Luc2 metastasis nodules in each group mice from panel **f**. **h** Representative images of lung metastasis nodules of 4T1-Luc2 and corresponding H&E staining of lungs from panel **f**. **i** *Hectd3* KO decreased lung metastasis of B16-F10 melanoma tumor cells. B16-F10 cells were injected by tail vein into WT (n=4) and *Hectd3*^-/-^ (n=5) C57BL/6 mice (1×10^5^ per mouse) which were pretreated with LPS (1 mg/kg) intravenous injection for 5 h. The mice were sacrificed 20 days after the tumor cell injection. Graph shows the number of pulmonary metastasis nodules in each group mice. **j** The weight of whole lung with B16-F10 metastasis nodules in each group mice from panel **i**. **k** Representative images of lung metastasis nodule of B16-F10 and corresponding H&E staining of lung from panel **i**. **l** WT and Hectd3^-/-^ BALB/c mice were intravenously injected with or without TNFα (200 μg/kg). Five hours later, 4T1-Luc2 cells were injected through the tail vein (2×10^5^ cells per mouse). The mice were sacrificed 20 days after injection of tumor cells. The graph shows the number of pulmonary metastasis nodules in each group of mice. **m** The weight of the whole lung with metastatic nodules in each group of mice from panel **l**. **n** Representative H&E staining of the lungs in different groups of mice from panel **l**.

Data are presented as mean ± SEM, and statistics were calculated using two-tailed t-test for **a-e**, **f**, **g**, **i** and **j**, two-way ANOVA for **l** and **m**. *, *P* < 0.05; **, *P* < 0.01; ***, *P* < 0.001; n.s., not significant. Scale bars, 2 mm for **h**, **k** and **n**.


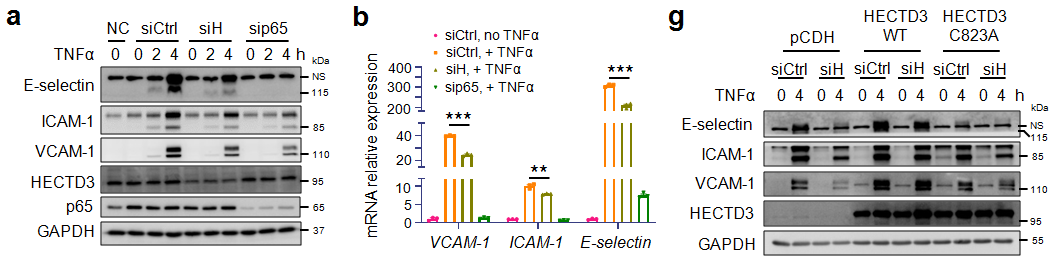


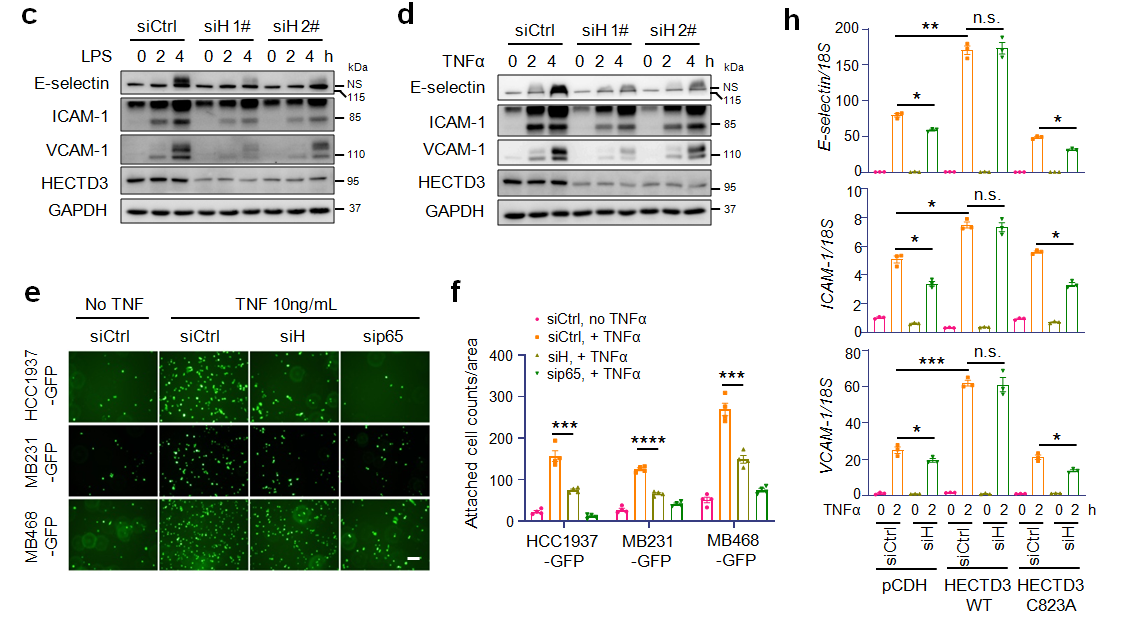

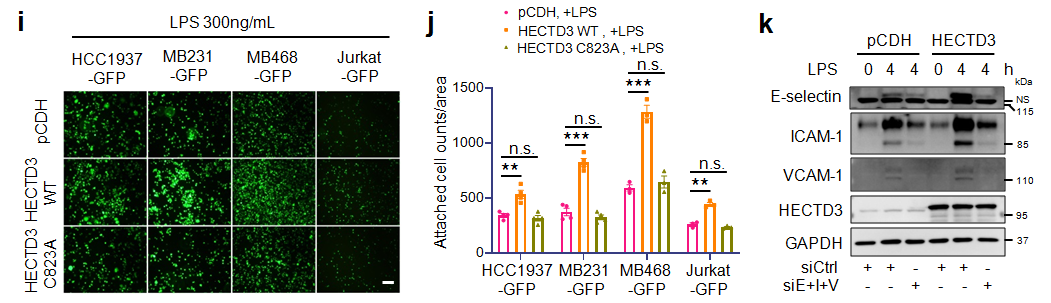


Figure. S2 HECTD3 promotes the adhesion of tumor cells to HUVECs by upregulating E-selectin, ICAM-1 and VCAM-1 expression in HUVECs. a HECTD3 knockdown decreased TNFα-induced protein expression of adhesion molecules, including E-selectin, ICAM-1 and VCAM-1 in HUVECs. HECTD3 or p65 were knocked down using corresponding siRNA for 36 h. HUVECs were stimulated with or without TNFα (10 ng/ml) for 0-4 h. siControl (siCtrl) was used as a negative control. sip65 was used as a positive control. siHECTD3 (siH) was a siRNA pool containing siHECTD3 1# and 2#. b HECTD3 knockdown decreased TNFα-induced mRNA expression of adhesion molecules, including E-selectin, ICAM-1 and VCAM-1 in HUVECs. qRT-PCR was used to analyze the mRNA levels of adhesion molecules. TNFα (10 ng/ml) stimulated the cells for 2 h. c HECTD3 knockdown decreased LPS-induced protein expression of adhesion molecules, including E-selectin, ICAM-1 and VCAM-1 in HUVECs. HECTD3 was knocked down using two different siRNAs for 36 h. HUVECs were stimulated with or without LPS (300 ng/ml) for 0-4 h. d HECTD3 knockdown decreased TNFα-induced protein expression of adhesion molecules, including E-selectin, ICAM-1 and VCAM-1 in HUVECs. HECTD3 was knocked down using two different siRNAs for 36 h. HUVECs were stimulated with or without TNFα (10 ng/ml) for 0-4 h. e Representative images of the adhesion of GFP-labeled tumor cells to monolayer-cultured HUVECs transfected with the indicated siRNA and stimulated with or without TNFα. Scale bars, 200 μm. f Bar graphs show the number of GFP-labeled tumor cells attached to monolayer-cultured HUVECs of panel e. g Exogenous HECTD3 increased TNFα-induced protein expression of adhesion molecules, including E-selectin, ICAM-1 and VCAM-1 in HUVECs in an E3 ligase activity dependent manner. HUVECs stably overexpressed siRNA-resistant HECTD3, C823A mutant, and control were established. These HUVECs were transfected with siControl or siHECTD3 respectively and stimulated with TNFα (10 ng/ml) for 4h. h Exogenous HECTD3 increased TNFα-induced mRNA expression of adhesion molecules, including E-selectin, ICAM-1 and VCAM-1 in HUVECs in an E3 ligase activity dependent manner. qRT-PCR was used to analyze the mRNA levels of adhesion molecules. TNFα (10 ng/ml) stimulated the cells for 2 hours. Endogenous HECTD3 was silenced by siRNA. i Representative images of the adhesion of tumor cells (HCC1937-GFP, MDA-MB-231-GFP, MDA-MB-468-GFP and Jurkat-GFP) to monolayer-cultured HUVECs transfected with HECTD3 (WT, C823A, and vector control) and stimulated with LPS (300ng/mL, 4h). j HECTD3 overexpression increased LPS-induced adhesion between tumor cells and HUVECs in an E3 ligase activity dependent manner. Bar graphes show the number of HCC1937-GFP, MDA-MB-231-GFP, MDA-MB-468-GFP and Jurkat-GFP cells attached to monolayer-cultured HUVECs of panel i. k HECTD3 increased LPS-induced tumor cell adhesion to HUVECs through upregulation the expression of adhesion molecules, including E-selectin, ICAM-1 and VCAM-1 in HUVECs. E-selectin, ICAM-1 and VCAM-1 were simultaneously knocked down by siE+I+V, a siRNA mixture of siE-selectin, siICAM-1 and siVCAM-1.

Data are presented as mean ± SEM, and statistics were calculated using two-tailed t-test for **b**, **f**, **h** and **j**. *, *P* < 0.05; **, *P* < 0.01; ***, *P* < 0.001; n.s., not significant. Scale bars, 200 μm for **e** and **i**.

**
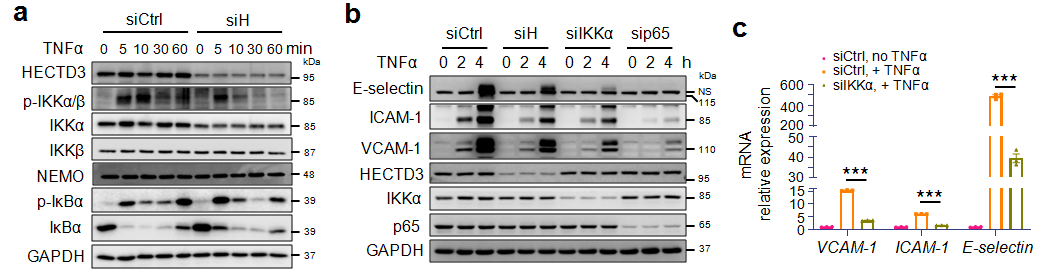

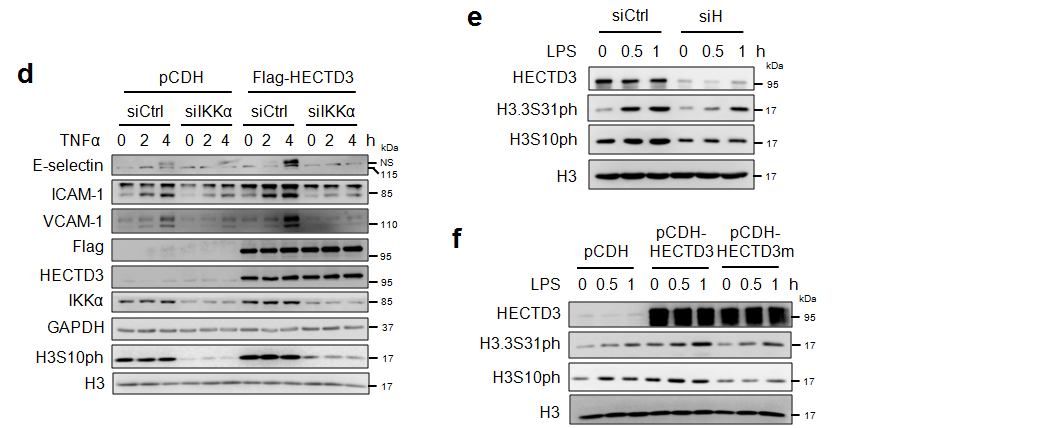
**


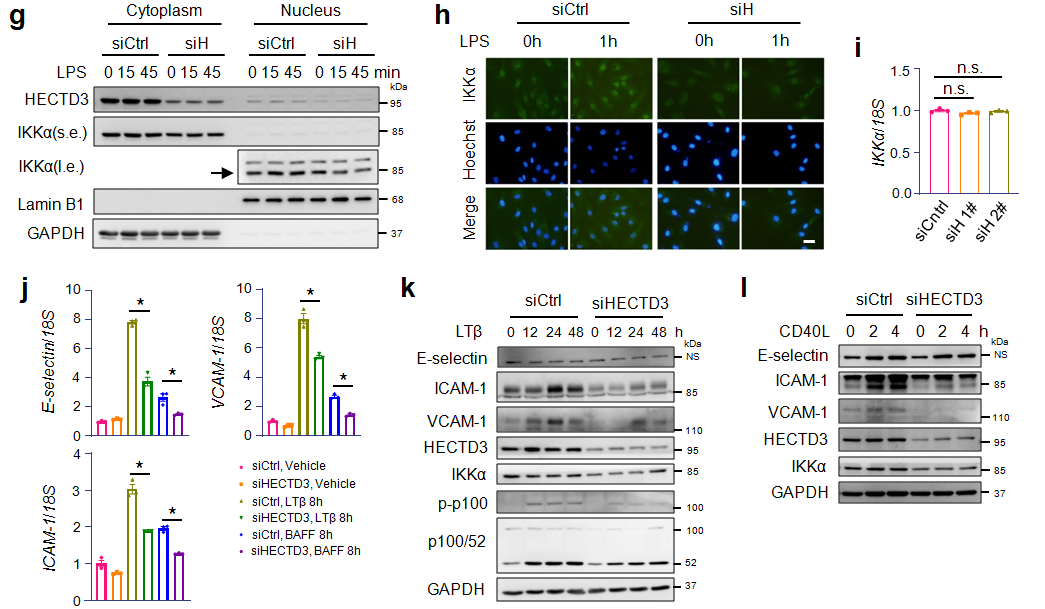


Figure. S3 HECTD3 increases the expression of adhesion molecules by stabilizing IKKα and recruiting nuclear IKKα to adhesion molecule gene promoters. a HECTD3 transient knockdown decreased the IKKα protein levels in HUVECs. HECTD3 was knocked down with an siRNA pool in HUVEC cells for 36 h. Following that, the cells were stimulated with TNFα (10 ng/ml) as indicated time (0-60 mins). b IKKα knockdown decreased TNFα-induced protein expression of adhesion molecules, including E-selectin, ICAM-1 and VCAM-1 in HUVECs. IKKα was knocked down using siRNA for 36 h. HUVECs were stimulated with or without TNFα (10 ng/ml) for 0-4 h. NS, nonspecific band. c IKKα knockdown decreased TNFα-induced mRNA expression of adhesion molecules, including E-selectin, ICAM-1 and VCAM-1 in HUVECs. IKKα was knocked down using siRNA for 36 h. HUVECs were stimulated with or without TNFα (10 ng/ml) for 2 h. d HECTD3 functions through IKKα to promote adhesion molecule expression in HUVECs. IKKα was transiently knocked down in HECTD3 overexpressing HUVECs. HUVECs were stimulated with or without TNFα (10 ng/ml) for 0-4 h. NS, nonspecific band. e Immunoblot analysis of H3.3S31ph and H3S10ph in HUVECs pretreated with LPS(300ng/mL) after knockdown of HECTD3. f Immunoblot analysis of H3.3S31ph and H3S10ph in HUVECs overexpressed HECTD3 or its catalytically inactive mutant with LPS treatment. g HECTD3 transient knockdown decreased the IKKα protein nuclear localization in HUVECs. HUVECs transfected with siRNA were stimulated with LPS (300 ng/ml) for 0-45 min. Nuclear and cytoplasmic fractions were isolated using the NE-PER Nuclear and cytoplasmic Extraction Reagents (Thermo scientific). s.e. and l.e. mean short exposure and long exposure, respectively. h HECTD3 transient knockdown decreased the IKKα protein nuclear localization in HUVECs. Immunofluorescence analysis of the subcellular localization of IKKα. Nucleus was stained by Hoechst. i HECTD3 did not affect *IKKα* mRNA levels in HUVECs. qRT-PCR was used to measure the *IKKα* mRNA levels in HUVECs knocking down HECTD3 with two different siRNA. 18S was used as internal control. j qRT-PCR analysis of adhesion molecules in HUVECs knocking down HECTD3 and stimulated with LTβ (50ng/mL,R&D, 8884-LY) or BAFF (100ng/mL, R&D, 2149-BF/CF) for 8 h. k Immunoblot analysis of adhesion molecules, like E-selectin, ICAM-1 and VCAM-1, and p100 processing in HUVECs knocking down HECTD3, and stimulated with or without LTβ (50ng/mL) as indicated time. NS, nonspecific band. l Immunoblot analysis of adhesion molecules, like E-selectin, ICAM-1 and VCAM-1 in HUVECs knocking down HECTD3, and stimulated with or without CD40L (500ng/mL, R&D, 6420-CL-025/CF) as indicated time. NS, nonspecific band.

Data represent 3 independent experiments for all of the above experiments. Data are presented as mean ± SEM, and statistics was perormed using two-tailed t-test for **c**, **i** and **j**. *, *P* < 0.05; ***, *P* < 0.001; n.s., not significant. Scale bars, 80 μm for **h**.

**
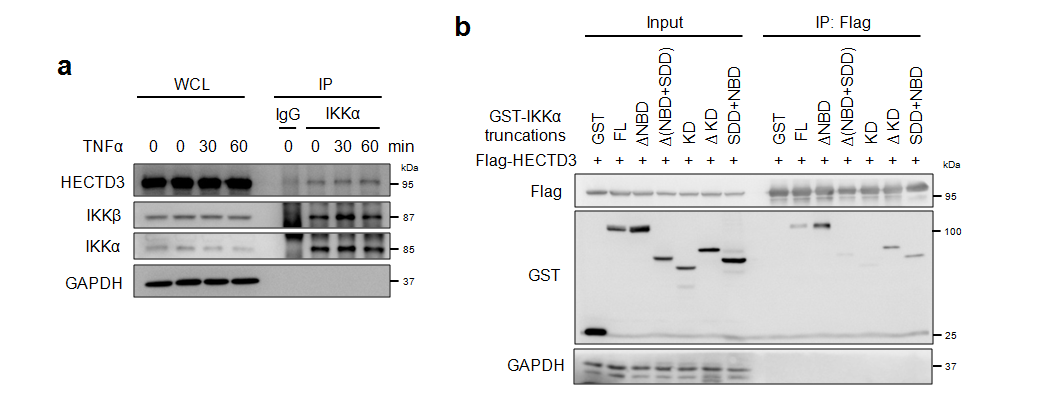
**

Figure. S4 HECTD3 interacts with IKKα. a TNFα did not regulate the interaction between HECTD3 and IKKα in HUVECs. HUVECs were stimulated with TNFα (10 ng/ml) for 0-1 h. Anti-IKKα antibody was used to Immunoprecipitate IKKα. The co-immunoprecipitated endogenous HECTD3 was unchanged. b The SDD domain of IKKα is responsible for the interaction with HECTD3. Flag- HECTD3 and GST-fused IKKα truncation mutants were co-expressed HEK293T cells. Flag-HECTD3 was immunoprecipitated with Flag-M2 beads, GST-IKKα KD and ΔNBD+SDD failed to interact with Flag-HECTD3, suggesting that SDD domain is important for the protein interaction.

Data represent 3 independent experiments for all of the above experiments.

**
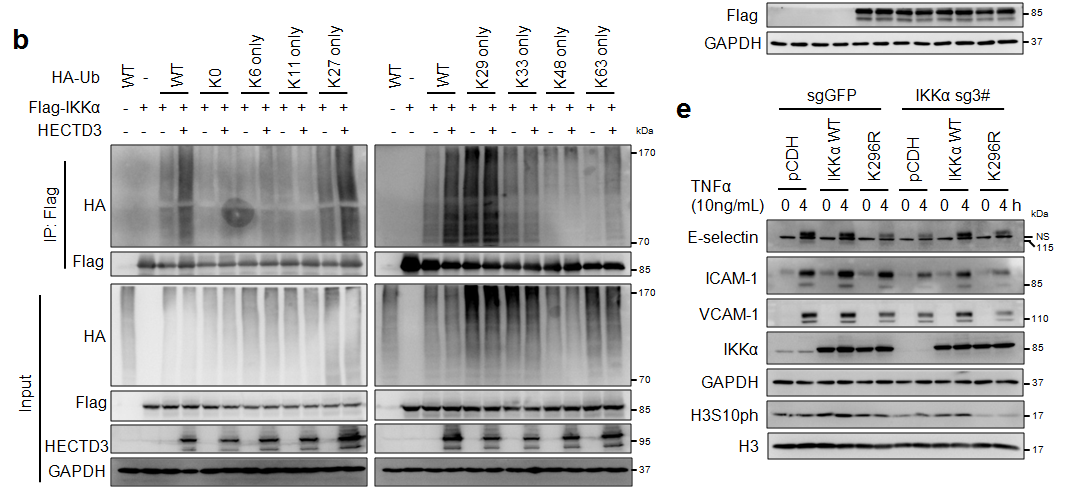

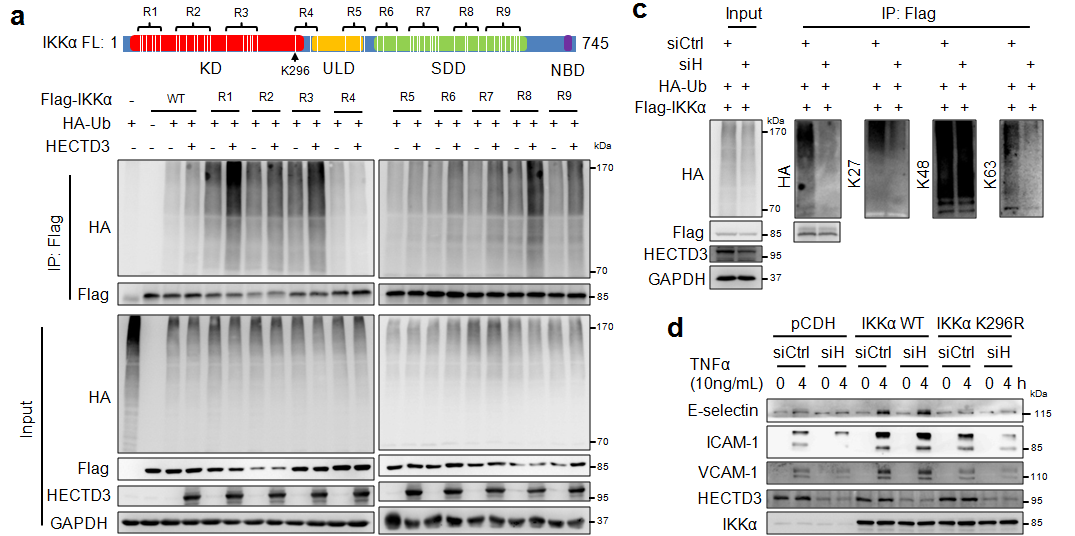

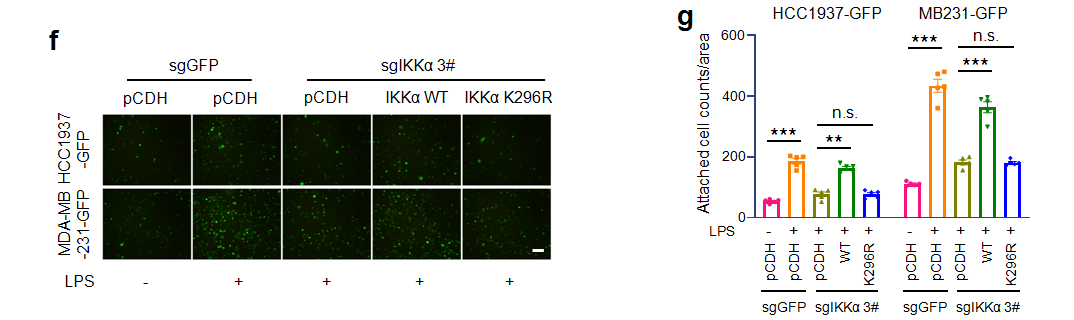

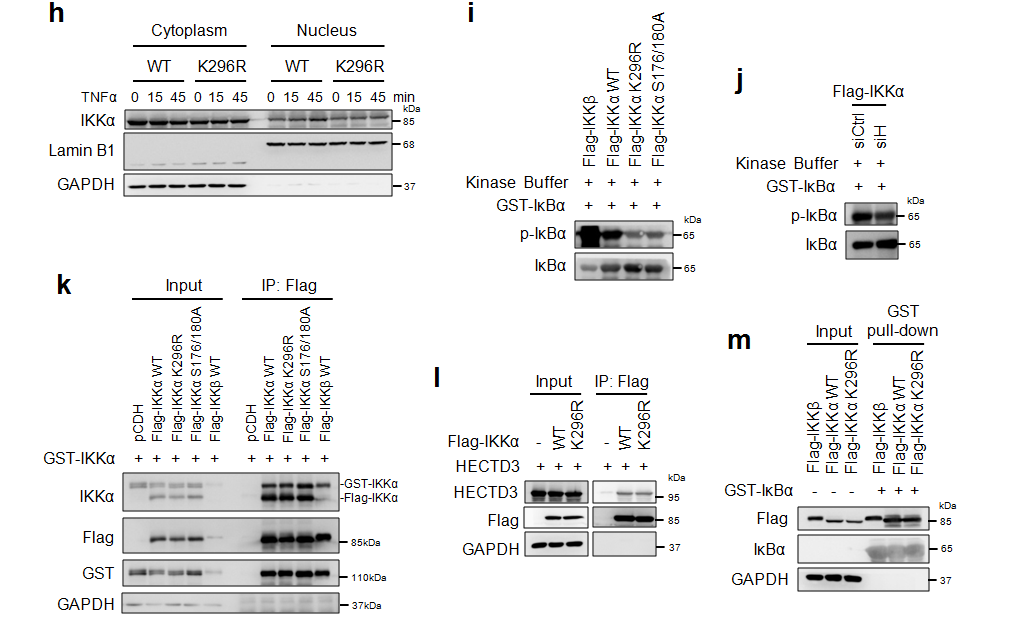

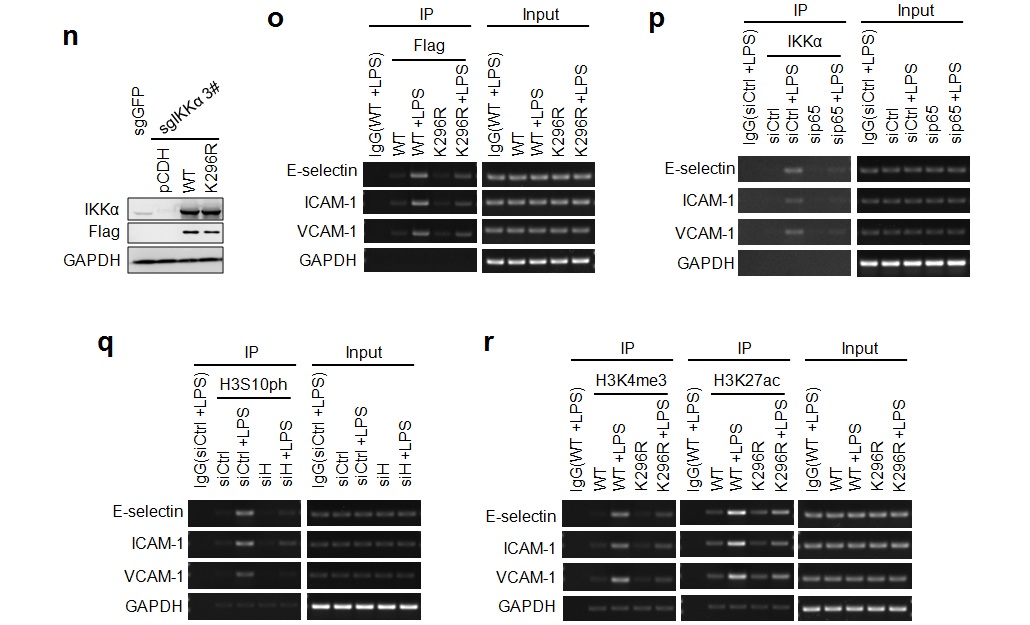
**

Figure. S5 HECTD3 ubiquitinates IKKα with the K27, K63-linked polyubiquitin chains at K296 and increases the IKKα protein stability and kinase activity. a HECTD3 ubiquitinates IKKα at K296 in HEK293T cells. Schematic diagram shows the regions of human IKKα artificially divided dependent on lysine residues distribution (top). HECTD3 failed to ubiquitinate Flag-IKKα R4, which contains K296, K311, K322 (bottom). b HECTD3 ubiquitinates IKKα with the K27- and K63-linked polyubiquitin chains. WT, K27 only, and K63 only HA-Ub supported HECTD3-mediated Flag-IKKα ubiquitination. In contrast, other HA–Ub mutants failed to do so. c HECTD3 ubiquitinates IKKα with the K27- and K63-linked polyubiquitin chains. Linkage-specific antibodies were used to validate the linkage of Flag-IKKα. HECTD3 knockdown decreased Flag-IKKα polyubiquitin chains were recognized by anti-HA, anti-K27, and anti-K63, but not anti-K48 antibody. d Flag-IKKα K296R failed to rescue HECTD3 knockdown induced adhesion molecule expression downregulation in response to TNFα. Flag-IKKα WT and Flag-IKKα K296R were stably overexpressed in HUVECs. HECTD3 knockdown dramatically blocked TNFα (10 ng/ml, 4h) induced adhesion molecule expression. Overexpression of Flag-IKKα WT completely rescued the HECTD3 knockdown induced phenotype. However, Flag-IKKα K296R failed to do so, suggesting that HECTD3 mediated IKKα ubiquitination at K296 is important for its functions. NS, nonspecific band. e Flag-IKKα K296R failed to phosphorylate histone H3 at Ser10 and induce adhesion molecule expression in HUVECs in response to TNFα. We generated an endogenous IKKα KO HUVECs using the CRISPR/Cas9 system. Unlike WT IKKα, IKKα K296R failed to phosphorylate histone H3 at Ser10 and induce adhesion molecule expression in HUVECs in response to TNFα (10 ng/ml, 4h). NS, nonspecific band. f Endogenous IKKα was stably knocked out in HUVECs using the CRISPR/Cas9 system. Restored the expression of IKKα by lentivirus encoding Flag-IKKα WT or Flag-IKKα K296R in these *IKKα* KO HUVEC cells to perform the *in vitro* adhesion assay with tumor cell lines HCC1937-GFP and MDA-MB231. g Bar graphs show the number of GFP-labeled tumor cells attached to monolayer-cultured HUVECs in f. h TNFα stimulation increased nuclear locolization of IKKα WT, not K296R, in HUVECs. Cytoplasmic and nuclear extracts of HUVECs overexpressing Flag-IKKα WT or K296R mutant and treated with TNFα were subjected to immunoblotting. i IKKα ubiquitination at K296 is essential for its kinase activity toward IκBα. The *in vitro* IKKα kinase assay contains purified Flag-IKKα WT, K296R, or S175/180A, GST-IκBα, and ATP. Flag-IKKα proteins were purified from HEK293T cells. Flag-IKKα, but not Flag-IKKα K296R, efficiently phosphorylate IκBα. j HECTD3 knockdown in HUVECs decreased the IKKα activity toward IκBα. After HECTD3 was knocked down, the purified Flag-IKKα decreased its kinase activity toward IκBα. k Flag-IKKα K296R did not change its dimerization in HEK293T cells. Flag-IKKα and GST-IKKα were co-expressed in HEK293T cells. Co-immunoprecipitation assays were performed using Flag-M2 beads. Both WT and K296R pulled down similar amount of GST- IKKα. l Flag-IKKα K296R still efficiently interacted with HECTD3, like WT Flag-IKKα. HECTD3 and Flag-IKKα WT or K296R were coexpressed into HEK293 cells and Flag-IKKα proteins were immunoprecipitated with Flag-M2 beads. m Flag-IKKα K296R did not affect the interaction with IκBα compared with Flag-IKKα WT. Cell lysates of HEK293T cells expressing Flag-IKKα WT or Flag-IKKα K296R were collected and incubated with purified GST-IκBα protein for 30 min on ice. The GST pull-down assay was performed using glutathione sepharose beads. n HUVECs stably knocked out endogenous IKKα using the CRISPR/Cas9 system was restored Flag-IKKα WT or K296R mutant. o Chromatin immunoprecipitation (ChIP) assays were performed using Flag-M2 beads in HUVECs knocked down p65 and stimulated with or without LPS (300 ng/mL) for 1 h. p ChIP assays were performed using anti-IKKα antibody in HUVECs form panel A and stimulated with or without LPS (300 ng/mL) for 1 h. q ChIP assays were performed using anti-pH3S10 in HUVECs knocked down p65 and stimulated with or without LPS (300 ng/mL) for 1 h. r ChIP assays were performed using anti-H3K4me3 or anti-H3K27ac in HUVECs form panel A and stimulated with or without LPS (300 ng/mL) for 1 h.

Data represent 3 independent experiments for all of the above experiments. Data are presented as mean ± SEM, and statistics were calculated using two-tailed t-test for **g**. **, *P* < 0.01; ***, *P* < 0.001; n.s., not significant. Scale bars, 200 μm for **f**.

**
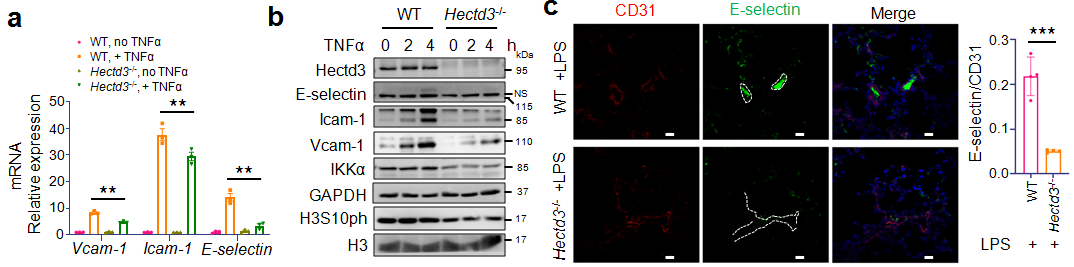

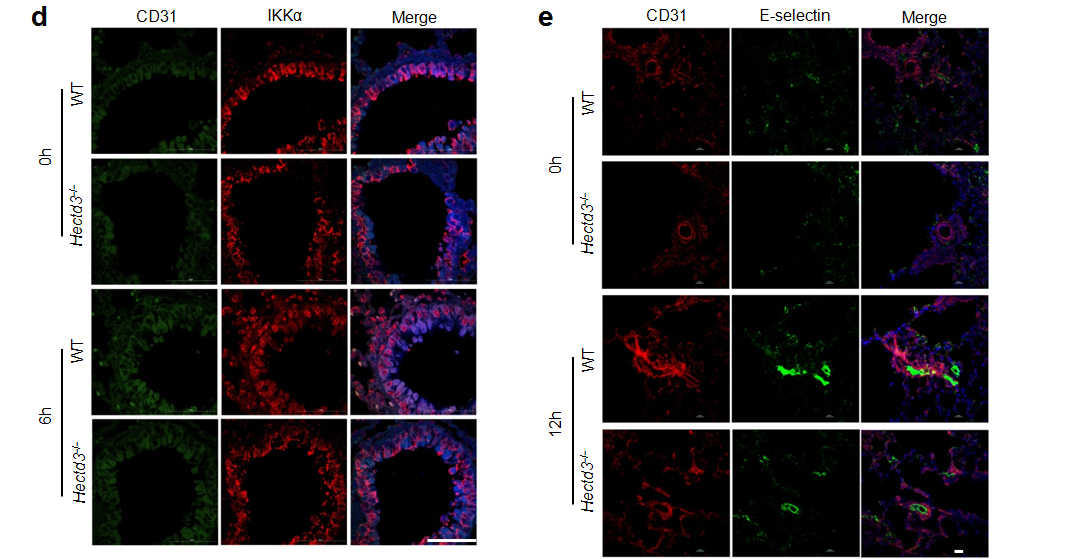

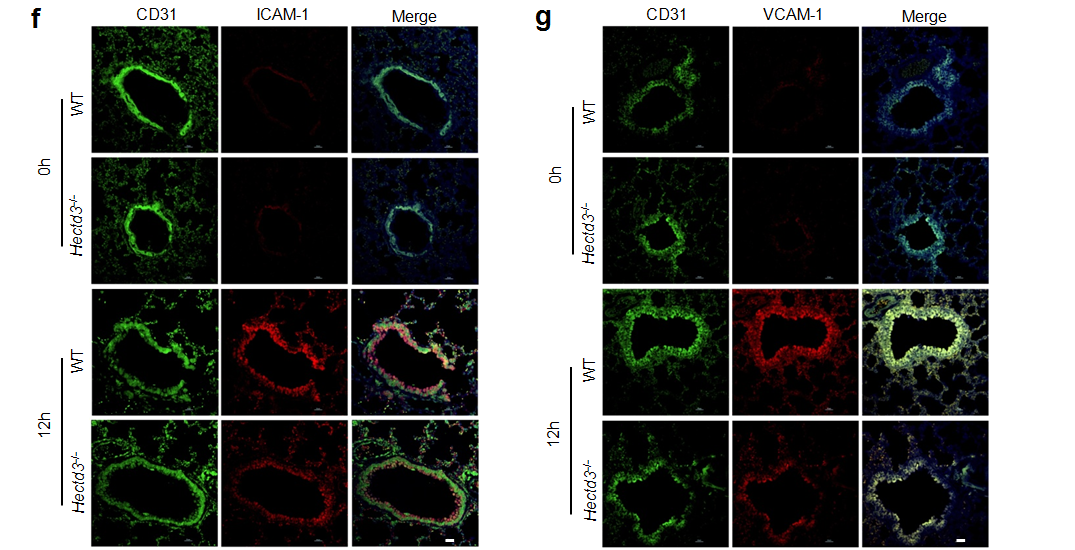
**
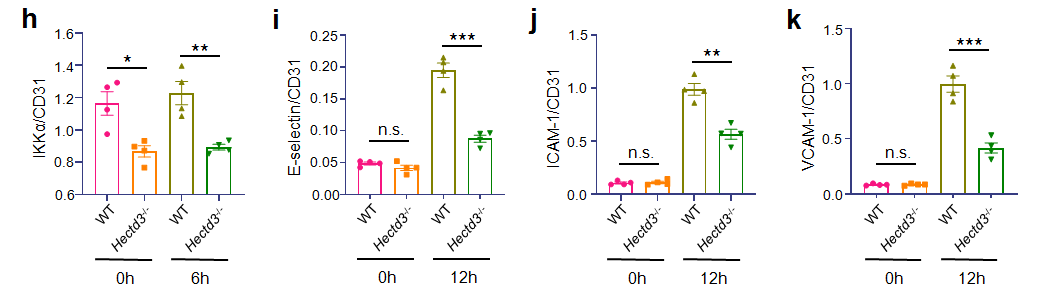

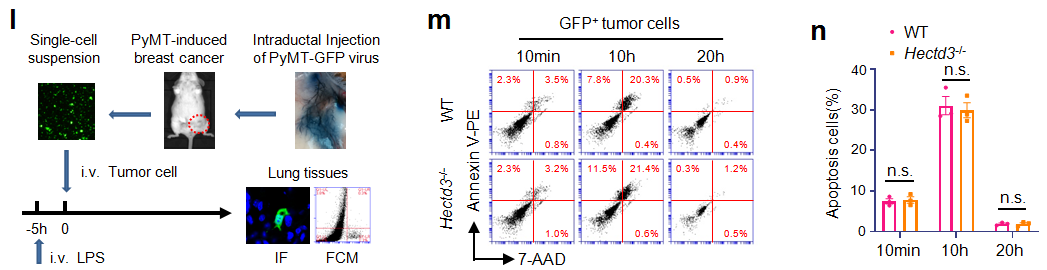

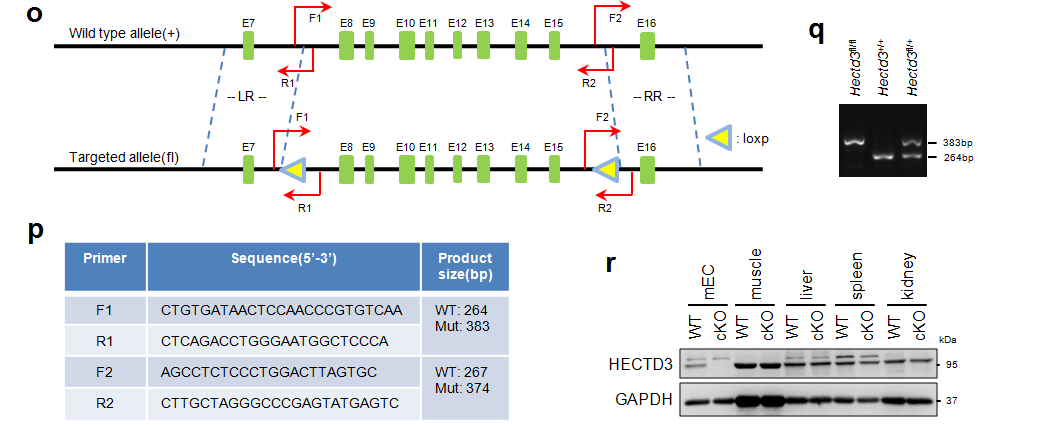

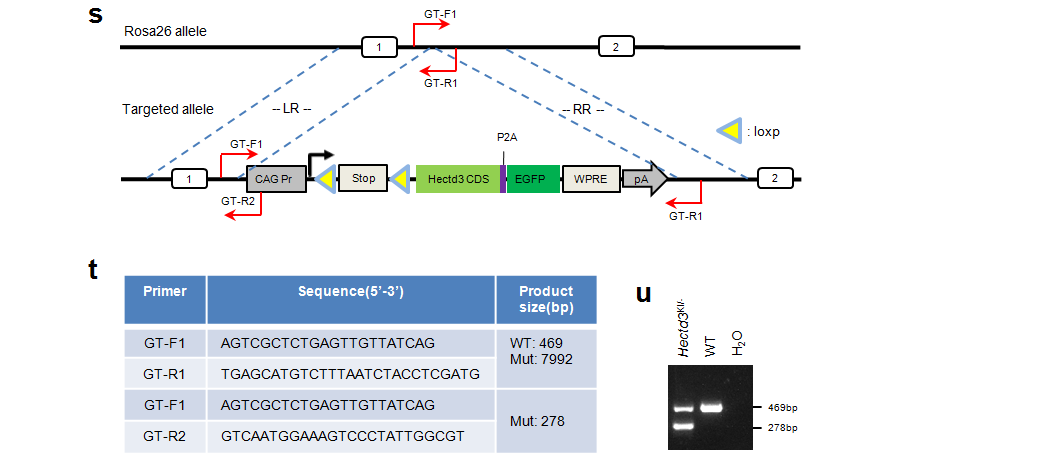

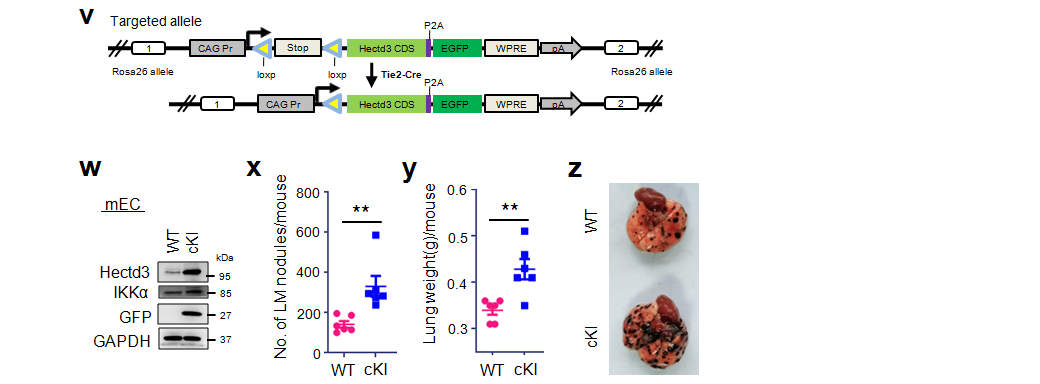


Figure. S6 Hectd3 promotes lung colonization of tumor cells under inflammatory conditions. a *Hectd3* KO significantly decreased TNFα-induced mRNA expression of adhesion molecules, including E-selectin, Icam-1 and Vcam-1 in mECs. qRT-PCR was used to analyze the mRNA levels of adhesion molecules. TNFα (10 ng/ml) stimulated the cells for 2 h. 18S rRNA was used as the internal control. b *Hectd3* KO significantly decreased TNFα-induced protein expression of adhesion molecules, including E-selectin, Icam-1 and Vcam-1 in mECs. IKKα and p-H3(Ser10) levels were also decreased in *Hectd3*^-/-^ mECs stimulated with TNFα (10 ng/ml) for 0-4 h. NS, nonspecific band. c WT or *Hectd3*^-/-^ mice was intravenously injected with LPS (1 mg/kg) for 5 h. Then the mice were sacrificed and perfused to analyze the adhesion molecules expression in the lung. Representative frozen immunofluorescence images of CD31 and E-selectin expressing vascular endothelial cells in lung (left), Scale bars, 20 μm. The ratio of the fluorescence intensity of E-selectin to that of CD31 is shown on the right. d 4T1-Luc2 cells were orthotopically transplanted to WT and Hectd3-/- BALB/c mice. Twelve days after transplantation, perfused and collected lungs of mice before (0h) or 6h after surgical resection of the primary tumors. Confocal microscopy images of CD31 and IKKα in lung tissues are shown. Scale bars, 50 μm. e Confocal microscopy images of CD31 and E-selectin in lung tissues of mice before (0h) or 12h after surgical resection of the primary tumors are shown. Scale bars, 20 μm. f Confocal microscopy images of CD31 and ICAM-1 in lung tissues of mice before (0h) or 12h after surgical resection of the primary tumors are shown. Scale bars, 20 μm. g Confocal microscopy images of CD31 and VCAM-1 in lung tissues of mice before (0h) or 12h after surgical resection of the primary tumors are shown. Scale bars, 20 μm. h The ratio of the fluorescence intensity of IKKα to that of CD31 is shown for panel d. i The ratio of the fluorescence intensity of E-selectin to that of CD31 is shown for panel e. j The ratio of the fluorescence intensity of ICAM-1 to that of CD31 is shown for panel f. k The ratio of the fluorescence intensity of VCAM-1 to that of CD31 is shown for panel g. l Schematic representation of the tumor cell colonization assay *in vivo*. 1) Lentiviruses overexpressing PyMT and GFP were injected intraductally to induce breast tumors. 2) Tumor cells were digested into a single-cell suspension. 3) The single-cell suspension (5×10^6^ cells per mouse) was injected through the tail vein into WT or *Hectd3*^-/-^ mice pretreated with LPS (1 mg/kg) stimulation for 5 h. 4) Twenty hours after tumor cell injection, the mice were sacrificed and perfused to analyze tumor cell colonization in the lung. m The GFP^+^ tumor cells (5×10^6^ cells per mouse) were injected through the tail vein into WT or *Hectd3*^-/-^ mice pretreated with LPS (1 mg/kg) stimulation for 5 h. Three mice for every group. At 10 minutes, 10 hours or 20 hours after tumor cell injection, the mice were sacrificed and perfused with PBS and the whole lungs were digested to cell suspension. Stained the cell suspension with Annexin V-PE and 7-AAD to analyze the apoptosis of GFP^+^ tumor cell in the lungs by FCM. n Quantified the percentage of apoptosis of GFP^+^ tumor cell in the lungs from panel m. o Targeting trategy used to generate Hectd3 flox mice. LR: left homologous arm, RR: right homologous arm. p Primers for genotyping of Hectd3 flox mice. q Genotyping of offspring generated from breeding of Hectd3^fl/+^ mice with primer F1 and R1. r The protein expression of Hectd3 in mECs isolated from the lungs, muscle, liver, spleen and kidney of *Tie2-Cre*^+^;*Hectd3*^wt^ (WT) and *Tie2-Cre*^+^;*Hectd3*^fl/fl^ (cKO) mice was detected by immunoblotting. s Targeting trategy used to generate Hectd3 knockin mice. The targeting sequence of CAG pr-loxP-Stop-loxP-Hectd3 CDS-P2A-eGFP-WPRE-pA into the Rosa26 site using the EGE system. LR: left homologous arm, RR: right homologous arm. t Primers for genotyping of Hectd3 KI mice. u Genotyping of offspring generated from breeding of *Hectd3*^KI/-^ mice primer GT-F1, GT-R1 and GT-R2. v Schematic representation of the creation of mice with Hectd3 conditional knockin (cKI) mice in endothelial cells. w The protein expression of Hectd3, IKKα and GFP in mECs isolated from the lungs of *Tie2-Cre*^-^;*Hectd3*^KI^ and *Tie2-Cre*^+^;*Hectd3*^KI^ mice was detected by immunoblotting. x B16-F10 cells were injected by tail vein into Tie2-Cre^-^;*Hectd3*^Ki^ and Tie2-Cre^+^;*Hectd3*^KI^ mice (1×10^5^ per mouse,6 mice/group) which were pretreated with LPS (1mg/kg) intravenous injection for 5 hours. The mice were sacrificed 20 days after the injection of cancer cells. The number of pulmonary metastasis nodules in each group of mice is shown. y Analysis of the weight of whole lung with metastasis nodules in each group mice from x. z Representative lung metastasis nodule images in each group mice from x.

Data are presented as mean ± SEM, and statistics were calculated using two-tailed t-test for **a**, **c**, **h**-**k**, **n**, **x** and **y**. *, *P* < 0.05; **, *P* < 0.01; ***, *P* < 0.001. n.s., not significant.

**
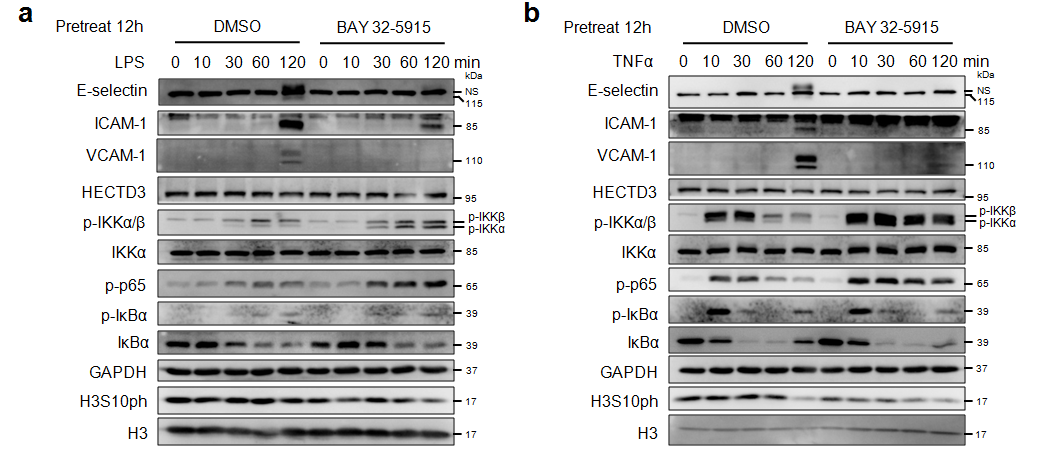

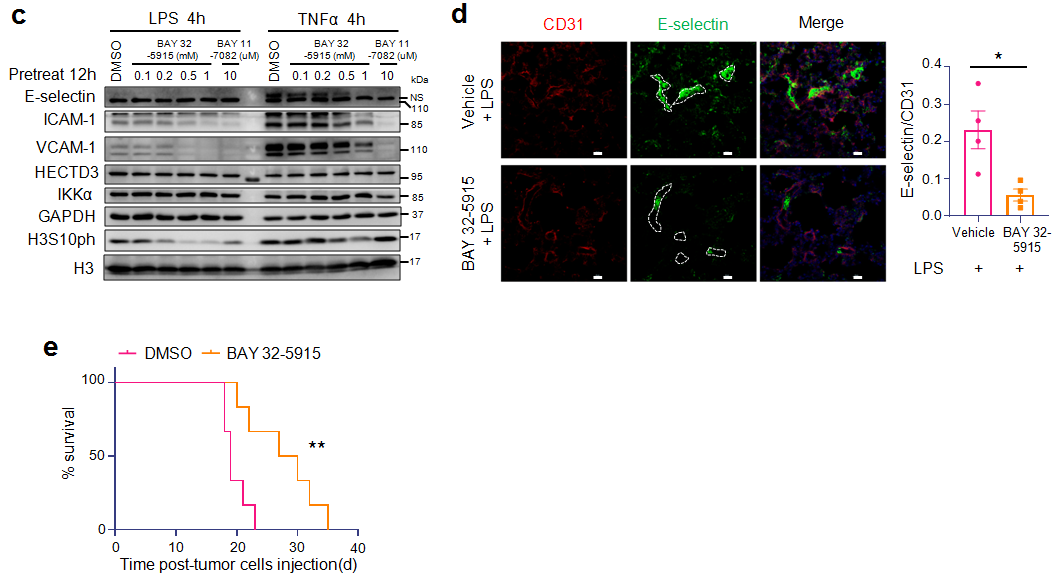
**

Figure. S7 BAY 32-5915, an IKKα kinase specific inhibitor, does not inhibit the IKK complex activation but blocks the targeted gene expression of NF-κB pathway. a BAY 32-5915 did not inhibit the IKK complex activation in response to LPS in HUVECs. HUVECs were pretreated with specific IKKα kinase inhibitor BAY 32-5915 (0.4 mM) or vehicle (DMSO) for 12 h, followed by the treatment of LPS (300 ng/ml) for 1-2 h. BAY 32-5915 did not inhibit the phosphorylation of IKKα/β, IκBα, and p65. The degradation of IκBα was also normal; however, the induction of adhesion molecules was dramatically blocked. The increase of the phosphorylation of IKKα/β and p65 by BAY 32-5915 might be caused by a negative feedback mechanism. NS, nonspecific band. b BAY 32-5915 did not inhibit the IKK complex activation in response to TNFα in HUVECs. HUVECs were pretreated with specific IKKα kinase inhibitor BAY 32-5915 (0.4 mM) or vehicle (DMSO) for 12 h, followed by the treatment of TNFα (10 ng/ml) for 1-2 h. NS, nonspecific band. c Immunoblot analysis of the expression of adhesion molecules in HUVECs pretreated with specific IKKα kinase inhibitor BAY 32-5915 as indicated concentration for 12 hours, DMSO as a negative control and BAY 11-7082(10μM) as a positive control, followed by treatment of LPS (300ng/mL) or TNFα (10ng/mL) for 4 hours. d Mice were pretreated with vehicle or BAY 32-5915 (25 mg/kg) for 24 h and LPS (1 mg/kg) for 5h by intravenous injection. Then the mice were sacrificed and perfused to analyze the adhesion molecule E-selectin expression in the lung. Representative frozen immunofluorescence images of CD31- and E-selectin expressing vascular endothelial cells in the lung are shown (left). Scale bars, 20 μm. The ratios of the fluorescence intensity of E-selectin to that of CD31 are shown on the right. e Kaplan-Meier survival curves of mice pretreated with DMSO (n=6) or IKKa inhibitor (BAY 32-5915) (n=6) for 24 h, followed by tail-vein injection of LPS and 4T1-Luc2 breast tumor cells.

Data represent 3 independent experiments for all of the above experiments. Data are presented as the mean ± SEM, and statistics was performed using a two-tailed t-test for **d**, and log-rank test for **e**. *, *P* < 0.05.
